# Biomimetic Exploration and Reflection on Switchable Coordination and Narrow‐Band Electrofluorochromic Devices

**DOI:** 10.1002/advs.202407219

**Published:** 2024-07-25

**Authors:** Baige Yang, Hengyuan Bai, Chenglong Li, Yu‐Mo Zhang, Sean Xiao‐An Zhang

**Affiliations:** ^1^ State Key Lab of Supramolecular Structure and Materials College of Chemistry Jilin University Changchun 130012 China

**Keywords:** biomimetic reflection, boron–nitrogen embedded polyaromatics, electrofluorochromic materials, fast switch, narrow band

## Abstract

Electrofluorochromic (EFC) materials and devices with controllable fluorescence properties show great application potential in advanced anticounterfeiting, information storage and display. However, the low color purity caused by the broad emission spectra and underperforming switching time of the existing EFC materials limit their application. Through biomimetic exploration and the study of reversible electrochemical responsive coordination reactions, boron–nitrogen embedded polyaromatics (B,N‐PAHs) with narrow‐band emission and high color purity have been successfully integrated into EFC systems, which also help to better understand the role of boron in biological activity. The EFC device achieve good performance containing quenching efficiency greater than 90% within short switching time (*t*_on_: 0.6 s, *t*_off_: 2.4 s), and nearly no performance change after 200 cycles test. Three primary color (red, green, and blue) EFC devices are successfully prepared. In addition, new phenomena are obtained and discussed in this biomimetic exploration of related boron reactions. The success and harvest of this exploration are expected to provide new ideas for optimizing properties and broadening applications of EFC materials. Moreover, it may provide ideas and reference significance for further exploring and understanding the function of boron compounds in biological systems.

## Graphical abstract

Here, an idea for designing narrow‐band EFC materials based on biomimetic electrochemically switchable coordination is utilized, and B,N‐PAHs are introduced into the EFC system. The EFC devices have been fabricated, which exhibit a narrow FWHM of 30 nm, a quenching efficiency greater than 90% within a short switching time (*t*_on_: 0.6 s, *t*_off_: 2.4 s).

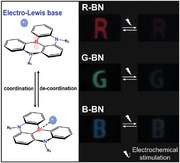

## 1 Introduction

Electrofluorochromic (EFC) materials have the ability of reversibly adjusting fluorescent properties under electrical stimulation.^[^ ^1^, ^2^, ^3^, ^4^, ^5^, ^6^ ^]^ They have gained increasing interest due to their potential application in emerging anticounterfeiting, information storage, display, etc.^[^ ^7^, ^8^, ^9^, ^10^, ^11^, ^12^ ^]^ Through the continuous efforts of researchers, two strategies have been reported to design EFC materials: one strategy involves electro‐switchable fluorophores, with many outstanding works reported, such as tetrazine derivatives,^[^ ^13^, ^14^, ^15^ ^]^ metal complex,^[^ ^16^, ^17^ ^]^ perylenediimide derivatives,^[^ ^18^ ^]^ triphenylamine derivatives,^[^ ^19^, ^20^, ^21^, ^22^ ^]^ boron dipyrromethene derivatives,^[^ ^23^, ^24^ ^]^ fluorane derivatives,^[^ ^25^, ^26^ ^]^ viologen derivatives,^[^ ^27^, ^28^, ^29^ ^]^ and so on, which can change the fluorescence properties by direct oxidation or reduction. The other is the molecular dyads formed by linking fluorophore to redox unit, and many interesting works have also appeared in this area. According to the type of redox active group, they can be divided into quinones‐based,^[^ ^30^, ^31^, ^32^, ^33^, ^34^, ^35^ ^]^ ferrocene‐based,^[^ ^36^, ^37^, ^38^ ^]^ tetrathiafulvalene‐based,^[^ ^39^, ^40^, ^41^ ^]^ triphenylamine‐based materials,^[^ ^42^, ^43^, ^44^ ^]^ and so on, which realizes fluorescence switching by electron transfer or energy transfer. Although a large number of new EFC compounds have been developed over the years, challenges remain for existing EFC materials, such as higher color purity and faster switching times (Table S1, Supporting Information), which are crucial to further promote the development and application of EFC materials, especially in the field of display that requires high color purity.

Boron–nitrogen embedded polyaromatics (B,N‐PAHs) with multiple resonance effects have made prominent research progress lately due to their narrow full‐width at half‐maximum (FWHM) and high color purity.^[^ ^45^, ^46^, ^47^, ^48^ ^]^ And a variety of new B,N‐PAHs have been developed for organic light‐emitting diode (OLED) materials with high color purity and potentially high luminescence efficiency.^[^ ^49^, ^50^, ^51^ ^]^ Their fascinating optical properties are mainly due to the excellent symmetry and rigid conjugated structure, and of course they are also related to the different electron‐donating/withdrawing abilities of the boron and nitrogen involved in the conjugation. It is precisely the synergistic effects of the two types of heteroatoms on the lowest unoccupied molecular orbital (LUMO) and the highest occupied molecular orbital (HOMO) and molecular polarization of the conjugated molecule that determine its optical properties. However, the reversibility and EFC performance of B,N‐PAHs in conventional electro‐redox reactions are still not ideal, which hinder their practical application in EFC system. Therefore, there is an urgent need to develop more effective strategies by leveraging the understanding of biological reaction mechanisms involving boron in nature to further enhance the comprehensive properties of boron‐embedded functional EFC materials. In addition, biomimetic exploration and harvest of this strategy of boron‐containing EFC functional materials and devices may also be helpful to further crack the magical working mechanism of trace element boron in life activities.

Although it is known that boron plays a magical role in the anti‐inflammatory, immune, nervous, and other biological processes in human organs,^[^ ^52^, ^53^, ^54^ ^]^ little is known about how boron participates in related life system activities. Especially, it is uncertain whether its working mechanism has certain similarities with the known chemical reversible regulation mechanism of boron embedded polyaromatics. Although it has been boldly speculated that the dynamic coordination process of the above boron‐containing compounds plays many important roles in living systems.^[^ ^55^, ^56^, ^57^ ^]^ However, the cruel fact is that this microdynamic process is complex and invisible, which makes it difficult to understand the internal mechanism and further exert its biological effects. If unconventional chemical methods can be used as tools to replace previously active invisible boric acid or boron ester derivatives in life activities with visible boron compounds with similar planar configurations and changes, and to visually explore their micro dynamic processes in organisms under electric stimulation, it might provide new insights and significant references for further understanding the dynamic reaction mechanisms of boron compounds in biological systems influenced by micro‐electric fields.

Here, an idea for designing narrow band EFC materials based on biomimetic electrochemically switchable coordination was utilized, and B,N‐PAHs were introduced into EFC system. Through a unique structural design, the EFC devices had been fabricated, which exhibited a narrow FWHM of 30 nm, a quenching efficiency greater than 90% within a short‐switching time (*t*_on_: 0.6 s, *t*_off_: 2.4 s), and nearly no noticeable performance degradation after 200 consecutive cycles. Meanwhile, EFC devices in three primary colors (red, green, and blue) were successfully prepared. Finally, the patterned, flexible, and anticounterfeiting protype devices based on this EFC system have been successfully fabricated and discussed in depth. Moreover, in‐depth exploration and discussion were conducted on the discovery of dynamic B–O coordination bonds involved in this biomimetic process, in order to better understand the role of boron compounds in organisms and microchemical reactions in biological electromagnetic field systems.

## 2 Results and Discussion

### 2.1 Design and Feasibility

As mentioned earlier, since the sp^2^‐hybrid boron atom in B,N‐PAHs act as good electron acceptor in conjugation,^[^ ^58^, ^59^, ^60^, ^61^, ^62^ ^]^ which makes it contribute a lot to the LUMO of the molecule. This means that chemical environment around boron atom has an effect on the optical properties of B,N‐PAHs. Therefore, we speculate on the following two strategies to influence charge density of boron to switch the fluorescence, which are expected to obtain excellent EFC properties of B,N‐PAHs.

**Figure 1:**
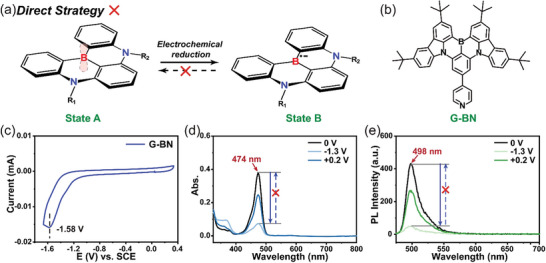
a) Schematic diagram of the direct redox strategy for introducing B,N‐PAHs into electrofluorochromic systems. b) Molecular structure of G‐BN. c) Cyclic voltammogram of G‐BN (1.0 × 10^–3^ mol L^–1^) in THF with 1.0 × 10^–1^ mol L^–1^ tetrabutylammonium hexafluorophosphate (TBAPF_6_). The spectra of d) absorption and e) emission spectra (ex = 465 nm) of the individual G‐BN (1.0 × 10^–4^ mol L^–1^) in THF with 1.0 × 10^–1^ mol L^–1^ TBAPF_6_ when the solutions were added 0, –1.3, and +0.2 V in situ, respectively. And the a.u. stands for arbitrary units.

The first strategy is the electrochemical reduction of electron‐deficient boron atom to switch its fluorescent properties, called direct redox strategy (**Figure** **1**a). In this work, G‐BN^[^ ^63^ ^]^ (Figure 1b and Figure S18, Supporting Information) with a FWHM of 21 nm as one of a simple structure for B,N‐PAHs was studied firstly as an example to prove our conjecture. As shown in Figure 1d,e, both the main visible absorption peaks (474 nm) and emission peak (498 nm) of G‐BN were decreased when the negative voltage (–1.3 V) was applied directly to G‐BN. This may be due to the chemical environment around boron atom was affected by the reduction reaction caused by electrochemical stimulation (Figure 1c). Unfortunately, the initial absorbance and fluorescence were not fully restored when opposite voltage (+0.2 V) was stimulated. Meanwhile, in situ measurement for the change of fluorescence at 498 nm and absorbance at 474 nm confirmed it (Figure S1, Supporting Information). The above results indicated that irreversible electrochemical side reactions occurred, which may be due to that the ionic radicals generated in the partial electro‐reduction reaction of G‐BN are not stable, and are prone to further intramolecular or intermolecular chemical reactions and structural changes. Clearly, these irreversible properties do not match the reversibility requirements of electrofluorescence. How to break the curse of coexistence of pros and cons, maintain its original advantages, and avoid the original shortcomings is a technological challenge that needs to be solved urgently.

**Figure 2:**
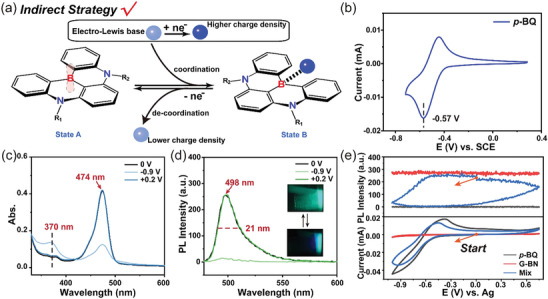
a) Schematic diagram of the indirect redox strategy for introducing B,N‐PAHs into EFC systems. b) Cyclic voltammogram of *p*‐BQ (1.0 × 10^–3^ mol L^–1^) in THF with 1.0 × 10^–1^ mol L^–1^ TBAPF_6_. The spectra of c) absorption and d) emission of the mixture of *p*‐BQ (1.0 × 10^–3^ mol L^–1^) and G‐BN (1.0 × 10^–4^ mol L^–1^) in THF with 1.0 × 10^–1^ mol L^–1^ TBAPF_6_ when the solutions were added 0, –0.9, and +0.2 V in situ, respectively. And the illustration is the actual photographs of this EFC system. e) Changes in emission spectra at 498 nm (top) during cyclic voltammograms (CVs, bottom) in situ of G‐BN (1.0 × 10^–4^ mol L^–1^), *p*‐BQ (1 × 10^–3^ mol L^–1^), and the mixture (mix) of G‐BN and *p*‐BQ (1.0 × 10^–4^ and 1.0 × 10^–3^ mol L^–1^) in THF with 1.0 × 10^–1^ mol L^–1^ TBAPF_6_, ex = 465 nm, and the a.u. stands for arbitrary units.

Considering the above consequences, the second strategy based on a biomimetic indirect redox method was proposed (**Figure** **2**a). The lack of local charge of boron atom makes it expected to interact with nearby external Lewis bases through e‐field activated Lewis acid–base pair.^[^ ^64^, ^65^ ^]^ This coordination is not only expected to indirectly affect the LUMO of the conjugated system, but also may change the three‐dimensional structure, degree of conjugation, and optical properties of the activated molecule by changing the valence state of boron atom. The above expected properties of B,N‐PAHs were experimentally verified chemically, which undergone a coordination reaction with the normal Lewis base (4‐dimethylaminopyridine, DMAP), accompanied by obvious changes in optical properties (Figure S2, Supporting Information). Therefore, we speculated that the fluorescent property of B,N‐PAHs could be switch reversibly if we adjusted the density of electric charge of Lewis base by using electrochemical stimulation, to switch coordination between sp^2^‐hybridized boron and Lewis base. And the materials with this property were defined as “electro‐Lewis base.”

Based on the above design, *p*‐benzoquinone (Coenzyme Q derivatives, *p*‐BQ) with good redox reversibility (Figure 2b) was chosen as the “electro‐Lewis base” due to its remarkable change of electric charge density before and after electro‐reduction.^[^ ^66^ ^]^ And the reduction voltage of *p*‐BQ at –0.57 V was much smaller than that of G‐BN at –1.58 V, the huge voltage gap between them ensured that G‐BN would not be reduced when *p*‐BQ can be reduced. In order to explore the feasibility of optical property change based on the electrochemical switchable coordination, the optical property of the mixture solution of G‐BN and *p*‐BQ was studied firstly. As shown in Figures 2c,d and S3 (Supporting Information), when a negative voltage (–0.9 V) was applied, both the absorbance and fluorescence changed obviously in the mixed solution. The emission peak at 498 nm and absorption peak at 474 nm of G‐BN both decreased significantly, while new absorption peak appeared at 370 nm and new emission peak appeared at 440 nm. Moreover, the above phenomenon was also consistent with the addition of sodium phenate, which acted as the mimic of product generated by electrochemical reduction of *p*‐BQ (Figure S4, Supporting Information). However, the absorbance and fluorescence of alone *p*‐BQ or G‐BN did not exhibit similar change under the same voltage (–0.9 V, Figure S5, Supporting Information). When an opposite voltage was applied, the optical properties of the mixed solution could return to the original state. This means that the mixture of G‐BN and *p*‐BQ emerged EFC property. In addition to G‐BN (498 nm), electrofluorescence of red and blue was successfully achieved when B‐BN (460 nm) and R‐BN (650 nm) were mixed with *p*‐BQ respectively (Figure S6, Supporting Information). Based on the above, the narrow band EFC system with three primary colors was successfully developed.

Meanwhile, the change of fluorescence at 498 nm and absorbance at 474 nm was studied by in situ electrochemical spectroscopy measurement, which can further prove that the EFC system was successfully prepared. As shown in Figures 2e and S7 (Supporting Information), changes in emission and absorption can only occurred when both *p*‐BQ and G‐BN were present, and they were fully reversible with the redox of *p*‐BQ, which proved that *p*‐BQ could reversibly adjust the structure of G‐BN. The above experimental results showed that B,N‐PAHs with high color purity were successfully introduced into EFC materials based on the electrochemical responsive coordination bonds developed in our previous work.^[^ ^67^ ^]^ The formation of the above coordination bond was further proved by ^1^H‐NMR with sodium phenolate (NaOC_6_H_5_) as a reference molecule added to G‐BN (Figure S8, Supporting Information). Moreover, the basic properties of fluorescence, such as fluorescence quantum efficiency (Ф_PL_) and fluorescence lifetime (τ), of the system in different states were characterized with sodium phenolate as mimic of product generated by the electro‐reduction of *p*‐BQ, and the results were listed in Table S2 (Supporting Information).

Based on the above experimental results and previous work,^[^ ^67^ ^]^ the mechanism of the reversible fluorescence switch can be outlined (Figure S9, Supporting Information). The initial state of B,N‐PAHs with narrow‐band emission was state A without any stimulation. When a negative voltage was applied, the *p*‐BQ was reduced to generate *p*‐BQ^2−^ with higher electron density, which can coordinate with B,N‐PAHs with Lewis acidity, accompanied by fluorescence changed to state B. When a positive voltage was applied, *p*‐BQ^2−^ was oxidized back to its initial state with lower electron density, causing it to de‐coordinate from B,N‐PAHs, and the fluorescence reappeared. The whole process was completely reversible. That is to say that the quenching of fluorescence of B,N‐PAHs was caused by the coordination between B,N‐PAHs and product generated by electro‐reduction of *p*‐BQ, resulting in boron atom of B,N‐PAHs from sp^2^ to sp^3^ hybridization, along with the change in conjugation structure, and the blue shift of absorption wavelength.

### 2.2 Reflection on Biomimetic Exploration of Boron‐Related Reaction in Organism

From this biomimetic exploration of the working mechanism of boron‐related chemicals in organism, several interesting new phenomena were discovered herein:

1) An electrochemically regulated dynamic coordination between B,N‐PAHs and *p*‐BQ was discovered and verified, which leaded to significant changes in optical properties. This phenomenon showed that the interaction between B,N‐PAHs and *p*‐BQ was the key to realize the change of optical properties. This phenomenon may also indirectly prove the interaction, functional, and dynamic bonding changes between borides in biological systems and coenzyme Q and/or amino acid residues (i.e., –(CH_2_)_4_–NH_2_, –COO^–^, etc.) in enzyme activity centers under bioelectrochemical regulation and related redox reactions (Figure S10, Supporting Information).
2) The chemically and electrochemically verified dynamic coordination between boron derivatives and coenzyme p‐BQ occurred after electrochemical stimulation. It was further confirmed that the coordination between them was carried out under e‐field. This seems to indicate that similar coordination and functional regulation of boron derivatives and coenzyme Q analogues may also possibly occur under the action of bioelectric fields.
3) Experimental data proved that the chemically/electrochemically activated free amine unit of lysine's derivative and carboxylic acid derivatives can dynamically coordinate with the boron derivatives and regulate their photophysical properties (Figure S10, Supporting Information), this also indicated that these amino acid residues may also be involved in the biological activity regulation of related enzymes under biological electric fields.

Based on the above experimental results and relevant considerations, we seem to have a better understanding and interpretation of the working mechanism of coenzyme Q and trace element boron in life activities as follows: It seems that the relevant ordered reaction processes are driven by local biological e‐fields. The e‐field causes coenzyme Q (or other amino acid residues, e.g., –(CH_2_)_4_–NH_2_, –COO^–^, etc.) to undergo a continuous reversible redox reaction and/or proton exchange to produces highly reactive electron‐rich molecular intermediates (coenzyme Q anion radicals or dianions or anion of other amino acid residues). For boron‐containing enzyme active centers, the resulting coenzyme Q anion radical or dianion and/or other active electron‐rich molecular intermediates can react dynamically with nearby boric acid or borate derivatives (or other amino acid residues). This process will lead to dynamic changes in their related supramolecular stereo‐structure and function composed of these amino acid residues around them, and promoting the continuous and orderly progress of related biological reactions.

### 2.3 Optimization of the EFC Device

**Figure 3:**
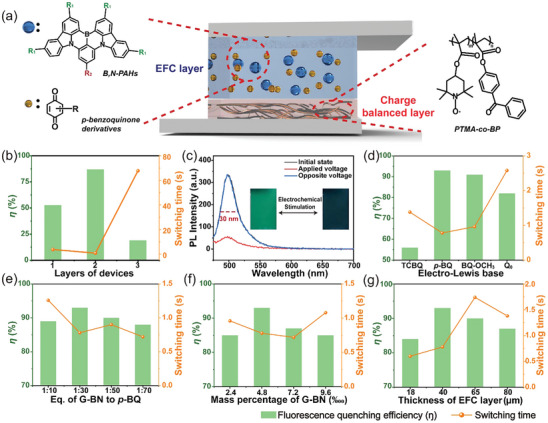
a) Schematic diagram of the e‐field‐driven fluorescence switching device structure. b) Optimization of device structure. c) Reversible fluorescence switch of the device in emission spectra when added voltages and its corresponding pictures, and the a.u. stands for arbitrary units. Optimization of the preparation parameters of the device: d) Types of “electro‐Lewis base”; e) Equivalent (Eq.) of G‐BN to *p*‐BQ; f) Mass percentage of G‐BN; g) Thickness of EFC layer (ex = 465 nm).

To further explore the potential application of this materials, we attempted to fabricate an EFC device containing B,N‐PAHs as the fluorescent molecule and *p*‐BQ as an “electro‐Lewis base.” However, the first challenge was that what type of EFC device structure was appropriate for this EFC material. In this case, following three types of devices containing G‐BN and *p*‐BQ were all constructed to explore the desired EFC performance, including a single‐layer gel device, a two‐layer gel device (**Figure** **3**a), and a three‐layer semi‐solid device. As shown in Figures 3b and S11 (Supporting Information), the fabricated three‐layer semi‐solid device exhibited less fluorescence quenching efficiency^[^ ^68^ ^]^ (*ŋ*, 19%), and a longer switching time (67.8 s). This is because the dynamic molecular motion of *p*‐BQ and G‐BN and the coordination/de‐coordination reaction between each other are severely hindered in semi‐solid environment. When the single‐layer gel device was used, a few fluorescence quenching efficiency (about 53%) can be achieved. This may because that quenching of fluorescence only occurs near the electrode for reduction reaction of *p*‐BQ, but it become invalid when near the electrode for oxidation reaction. Fortunately, the gel‐like EFC layer in two‐layer devices exhibited the faster switching rate (0.8 s) due to the more favorable environment for intermolecular reaction, and the more effective fluorescence quenching efficiency (87%) was measured. This two‐layer device structure consisting of a gel EFC layer and a charge balanced layer (PTMA‐co‐BP) was designed. Hereinto, PTMA‐co‐BP was immobilized on the surface of the ITO electrode by photo‐crosslinking technology, which had good solvent resistance.^[^ ^69^ ^]^ Two‐layer EFC devices with a FWHM of 30 nm (Figure 3c) brings high color purity, which is exactly needed in the display field. The initial state of the device exhibited strong fluorescent. After a negative voltage was applied, the fluorescence intensity at 498 nm decreased significantly and returned to the initial state quickly after the voltage was removed. This indicated that the fluorescence of the device can be switched reversibly.

In order to obtain the optimal performance of EFC devices, the important parameters involved in the fabrication process were considered systematically. Two performance indicators were used to assess the EFC performance, containing the *ŋ*, and switching time which is the time at the 90% of the full fluorescence change occurs after applying potential (Scheme S6, Supporting Information). Based on the mechanism of intermolecular electrochemically switchable coordination, the electron density of “electro‐Lewis base” is very important for the EFC switching of devices. Several *p*‐BQ derivatives with different substituents were chosen (Figures 3d and S12, Supporting Information). With the enhancement of electron‐donating ability of “electro‐Lewis bases,” *ŋ* increased by comparison with tetrachloro‐1,4‐benzoquinone (TCBQ) and *p*‐BQ. However, when the electron‐donating ability enhanced further, such as 2‐methoxy‐1,4‐benzoquinone (BQ‐OCH_3_) and 2,3‐dimethoxy‐5‐methyl‐1,4‐benzoquinone (Q_0_), the *ŋ* showed a decreasing trend. This may be because the steric hindrance of substituents makes coordination change more difficult. Therefore, *p*‐BQ with suitable electron‐donating ability was chosen as “electro‐Lewis bases” to fabricate the further EFC devices.

Since the fluorescent switch is due to the interaction between *p*‐BQ and G‐BN, there is no doubt that adjusting the equivalent (Eq.) of *p*‐BQ relative to G‐BN would affect switching performance. As shown in Figures 3e and S13 (Supporting Information), the *ŋ* increased with the increase of *p*‐BQ equivalent. But excess *p*‐BQ would quench the initial fluorescence of G‐BN, resulting in a lower *ŋ* from applying voltage. Therefore, with the increase of *p*‐BQ equivalent, the *ŋ* showed a trend of increasing first and then decreasing. And 30 equivalent of *p*‐BQ relative to G‐BN was best parameter. Then, the content of G‐BN in EFC layer was explored as shown in Figures 3f and S14 (Supporting Information). As the concentration of G‐BN increased from 2.4‱ (wt‱) to 4.8‱ (wt‱), the *ŋ* increased. However, when the content of G‐BN continued to increase, *ŋ* decreased. This phenomenon may be due to the aggregation induced quenching of G‐BN at high concentration in EFC layer. Therefore, the content of G‐BN was fixed at 4.8‱ (wt‱) in the further optimization. Finally, the influence of the thickness of EFC layer was investigated (Figures 3g and S15, Supporting Information). As the increase of the thickness, the *ŋ* and switching time both increased first and then decreased. After comprehensive consideration, the thickness of EFC layer was finally selected as 40 µm.

### 2.4 EFC Performance of Devices

**Figure 4:**
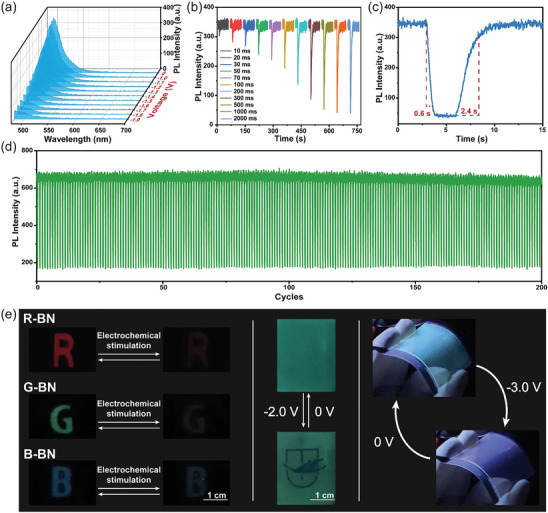
a) Emission spectra of the EFC device stimulated with different voltages for 3 s (ex = 465 nm). b) Fluorescence at 498 nm of the EFC device stimulated with −1.9 V for different times (ex = 465 nm). c) Switching times of the EFC device stimulated with −1.9 and +1.0 V (ex = 465 nm). d) Stability of the device at 498 nm under 200 cycles, stimulated with −1.25 V 8 s and +0.9 V 5 s. And the a.u. stands for arbitrary units. e) The prototypes for three‐primary display, patterned display and flexible display (ex = 365 nm).

The basic properties of EFC devices after optimizing were tested. As shown in **Figure** **4**a, the EFC switching was related to voltage. When –1.0 V was applied, the fluorescence intensity began to decrease. And when –1.9 V was applied, the *ŋ* was greater than 90%. Meanwhile, the fluorescence intensity of device changed in a gradient with the stimulation time (Figure 4b). What's more, thanks to the unique device structure, EFC device exhibited a surprising switching time. It took only 0.6 s to quench 90% of the initial state, and 2.4 s to recover to 90% of the initial state (Figure 4c). At the same time, B,N‐PAHs derivatives (B‐BN: 460 nm and R‐BN: 650 nm) have also been successfully prepared into EFC devices (Figure S16, Supporting Information), which proved the universality of the proposed electric switching coordination bond, and successfully prepared three primary color EFC devices, and provided more possibilities for the subsequent applications. Moreover, after 200 cycles of continuous switching tests, the performance of the EFC device showed almost no significant change (Figure 4d). Finally, as shown in Figure 4e, in order to directly exhibit the potential application of EFC devices, the three‐primary display and pattern display were achieved. And due to the inherent flexibility of EFC materials, they have application prospects in flexible displays. In the case of bending device, fluorescence switching could still be realized well. And at the quenched state, the blue fluorescence was due to the intrinsic fluorescence of PET‐ITO (Figure S17, Supporting Information).

## 3 Conclusion

In summary, we successfully introduced boron‐nitrogen embedded polyaromatics (B,N‐PAHs) with narrow‐band emission into EFC system based on the biomimetic reaction mechanism of electrochemically responsive coordination bond. This EFC device exhibited narrow FWHM and high color purity, and had good EFC properties, such as more than 90% quenching efficiency within short switching time (*t*_on_: 0.6 s, *t*_off_: 2.4 s) and good cycle stability (>200 cycles). Additionally, EFC devices in the three primary colors (red, green, and blue) were successfully prepared. And the application of those narrow‐band EFC materials and devices in display were also exploited. We believe that the proposal of this new biomimetic reaction mechanism will open a way for the performance optimization of EFC materials, and will provide more possibilities for the practical application of EFC materials. Moreover, the newly discovered and verified biomimetic dynamic bond may offer valuable insights and references for the in‐depth study and understanding of how boron compounds function in biological systems.

## 4 Experimental Section

### Electrochemistry

A three‐electrode system was used for cyclic voltammograms measurement, including a glass–carbon working electrode (3 mm dia.), a Pt wire counter electrode and an Ag wire reference electrode. A three‐electrode system was used for spectro‐electrochemistry measurement, including a Pt net as the working electrode, a Pt wire as the counter electrode, and an Ag wire electrode as the reference electrode. The more details are listed in the supporting information.

### Fabrication of the EFC devices

*Fabrication of Two‐Layer Devices with G‐BN/B‐BN/R‐BN as EFC Material*: EFC solution: PMMA (24.7%, wt%), TBAPF_6_ (1.1%, wt%), 1,4‐dutyrolactone (73.9%, wt%), electro‐Lewis base (*p*‐BQ or TCBQ or BQ‐OCH_3_ or Q_0_) (0.252%, wt%) and *G‐BN/B‐BN/R‐BN* (0.048%, wt%) in solvent.

*Ion storage solution: PTMA‐co‐BP (10 mg/mL) in THF*: First, the EFC layer was deposited by drop coating on the first ITO glass in the glove box. Next, the ion storage film was deposited by spin coating on the second ITO glass. Then, the ion storage layer was obtained from UV‐crosslinking in the glove box (254 nm for 10 min). Finally, two‐layer EFC device was fabricated by assembling the two ITO glasses together.

*Fabrication of One‐Layer Devices with G‐BN as EFC Material*: EFC solution: PMMA (24.7%, wt%), TBAPF_6_ (1.1%, wt%), 1,4‐dutyrolactone (73.9%, wt%), *p*‐BQ (0.252%, wt%), G‐BN (0.048%, wt%) and PTMA‐co‐BP (10 mg/mL) in tetrahydrofuran (THF).

The EFC layer was deposited by drop coating on the first ITO glass in the glove box. Then, a single‐layer device was obtained with assembling the second ITO glass on the EFC film.

*Fabrication of Three‐Layer Devices with G‐BN as EFC Material*: EFC solution: PMMA (59.1%, wt%), TBAPF_6_ (1.1%, wt%), 1,4‐dutyrolactone (39.5%, wt%), *p*‐BQ (0.252%, wt%) and G‐BN (0.048%, wt%) in THF.

Ion conductive solution: PMMA (60.2%, wt%), TBAPF_6_ (25.1%, wt%) and 1,4‐dutyrolactone (14.7%, wt%) in THF.

Ion Storage Solution: PTMA‐co‐BP (10 mg/mL) in THF.

First, the EFC layer was deposited by drop coating on the first ITO glass. Next, the ion storage film was deposited by spin coating on the second ITO glass. Then, the ion storage layer was obtained from UV‐crosslinking in the glove box (254 nm for 10 min). Then, the ion conductive film was obtained by continuing to drop the ion conductive solution on the ion‐storage film. Finally, three‐layer EFC device was fabricated by assembling the two ITO glasses together. The whole process took place in the glove box.

## Conflict of Interest

The authors declare no conflict of interest.

## Author Contributions

Y.‐M.Z., S.X.‐A.Z., and B.Y. conceived this project, designed the experiments, and wrote and revised the manuscript. The manuscript was written through contributions of all authors. B.Y. and H.B. performed the experiments. C.L. provided the G‐BN, B‐BN, and R‐BN. All authors have given approval to the final version of the manuscript.

## Supporting information

Supporting Information

## Data Availability

The data that support the findings of this study are available in the supplementary material of this article.
