# Supplementary material for: Biomimetic Exploration and Reflection on Switchable Coordination and Narrow‐Band Electrofluorochromic Devices
Source: Adv Sci (Weinh). 2024 Jul 25;11(36):2407219. doi: 10.1002/advs.202407219 (PMC11423134; doi:10.1002/advs.202407219)
Supplement: Supplementary file 1 — Supporting Information [file ADVS-11-2407219-s001.docx]

Supporting Information

Biomimetic Exploration and Reflection on Switchable Coordination and Narrow-band Electrofluorochromic Devices

*Baige Yang*^1^, *Hengyuan Bai*^1^, *Chenglong Li*^1^, *Yu-Mo Zhang**^,1^ and *Sean Xiao-An Zhang**^,1^

B. Yang, H. Bai, C. Li, Y.-M. Zhang, S. X.-A. Zhang
State Key Lab of Supramolecular Structure and Materials, College of Chemistry, Jilin University, Changchun, 130012, China
E-mail: seanzhang@jlu.edu.cn; zhangyumo@jlu.edu.cn

**Contents**

[Methods 3](#_Toc170411057)

[Method 1: Materials 3](#_Toc170411058)

[Method 2: Instrument characterization 3](#_Toc170411059)

[Method 3: Electrochemistry 3](#_Toc170411060)

[Method 4: Fabrication of the electrofluorochromic (EFC) devices 4](#_Toc170411061)

[Notes 7](#_Toc170411062)

[Figures 8](#_Toc170411063)

[Tables 26](#_Toc170411064)

[The References 31](#_Toc170411065)

# Methods

## Method 1: Materials

Poly(methyl methacrylate) (PMMA), 4-Dimethylaminopyridine (DMAP) were purchased from Aladdin Chemicals, China. Trifluoroacetic acid (CF_3_COOH), p-benzoquinone (*p*-BQ), tetrachloro-1,4-benzoquinone (TCBQ), 2,3-dimethoxy-5-methyl-1,4-benzoquinone (Q_0_), hydroquinone (HQ), 1,4-dutyrolactone, anisole, and ferrocene were purchased from Energy Chemicals, China. 2-methoxy-1,4-benzoquinone (BQ-OCH_3_) was purchased from TCI, China. B,N-PAHs (R-BN, G-BN and B-BN) were obtained in collaboration with professor Chenglong Li. PTMA-co-BP was prepared according to previous methods.^[S1]^ Tetrabutylammonium hexafluorophosphate (TBAPF_6_) was recrystallized for three times in anhydrous ethanol and dried under vacuum overnight before using. The indium tin oxide (ITO)-glass electrode was purchased from South China Xiang Science & Technology company.

## Method 2: Instrument characterization

UV-vis absorbance spectra and kinetic data were recorded using a Shimadzu UV-2600i PC double-beam spectrophotometer. Fluorescence spectra and kinetic data were obtained with a Shimadzu spectrofluorimeter RF-5301PC. Cyclic voltammograms were measured by Bio-logic electrochemical work station. The three-electrode cell, for cyclic voltammograms measurement, consisted of a glass-carbon working electrode (3 mm dia., Chenhua, China), a Pt wire counter electrode (Chenhua, China) and an Ag wire reference electrode (Chenhua, China). Thicknesses of electrofluorochromic layer were measured with digimatic micrometer from EXPLUIT.

## Method 3: Electrochemistry

As shown in **Scheme S1**, a three-electrode system was used for spectro-electrochemistry measurement, including a Pt net as the working electrode, a Pt wire as the counter electrode, and an Ag wire electrode as the reference electrode.


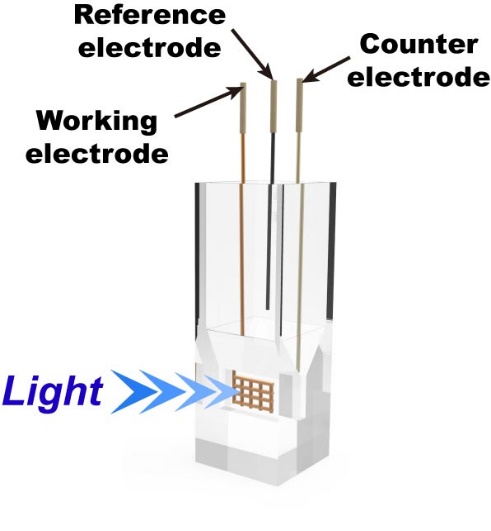


**Scheme S1.** Diagram of electrochemical cell which was used to measure spectro-electrochemistry in solution ‘in situ’.

As shown in **Scheme S2**, a three-electrode system was used for cyclic voltammograms measurement, including a glass-carbon working electrode (3 mm dia.), a Pt wire counter electrode and an Ag wire reference electrode.


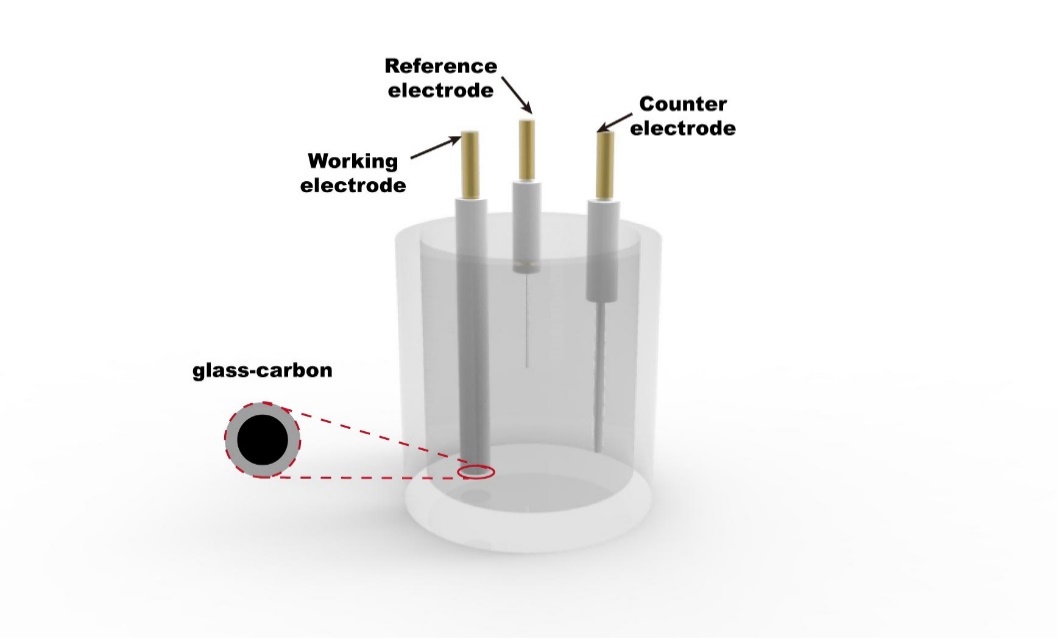


**Scheme S2.** Diagram of electrochemical cell which was used to measure cyclic voltammograms.

## Method 4: Fabrication of the electrofluorochromic (EFC) devices

**Fabrication of devices with G-BN as EFC material**

**Two-layer EFC devices**

EFC solution: PMMA (24.7%, wt%), TBAPF_6_ (1.1%, wt%), 1,4-dutyrolactone (73.9%, wt%), electro-Lewis base (*p*-BQ or TCBQ or BQ-OCH_3_ or Q_0_) (0.252%, wt%) and G-BN (0.048%, wt%) in tetrahydrofuran (THF).

Ion storage solution: PTMA-co-BP (10 mg/mL) in THF.

As shown in **Scheme S3**, first, the EFC layer was deposited by drop coating on the first ITO glass in the glove box. Next, the ion storage film was deposited by spin coating on the second ITO glass. Then, the ion storage layer was obtained from UV-crosslinking in the glove box (254 nm for 10 min). Finally, two-layer EFC device was fabricated by assembling the two ITO glasses together.


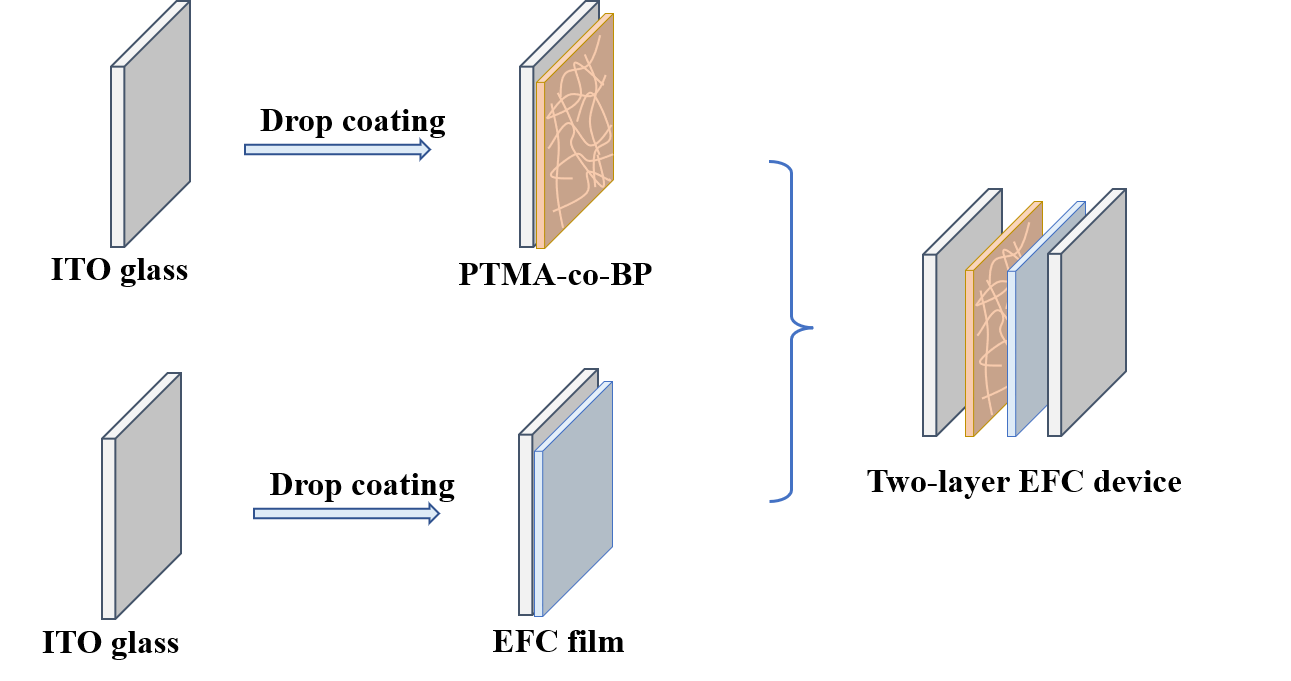


**Scheme S3.** Fabrication process of two-layer EFC device.

**One-layer EFC devices**

EFC solution: PMMA (24.7%, wt%), TBAPF_6_ (1.1%, wt%), 1,4-dutyrolactone (73.9%, wt%), *p*-BQ (0.252%, wt%), G-BN (0.048%, wt%) and PTMA-co-BP (10 mg/mL) in tetrahydrofuran (THF).

As shown in **Scheme S4**, the EFC layer was deposited by drop coating on the first ITO glass in the glove box. Then, a single-layer device was obtained with assembling the second ITO glass on the EFC film.


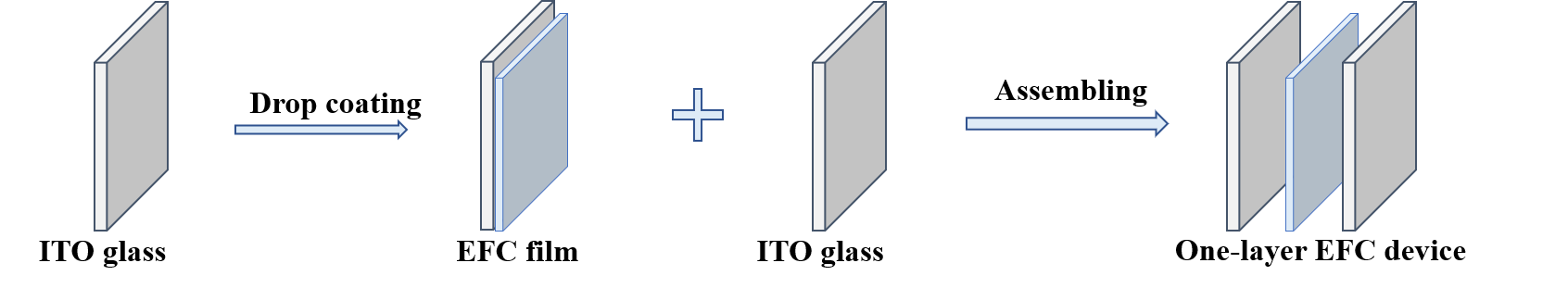


**Scheme S4.** Fabrication process of one-layer EFC device.

**Three-layer EFC devices**

EFC solution: PMMA (59.1%, wt%), TBAPF_6_ (1.1%, wt%), 1,4-dutyrolactone (39.5%, wt%), *p*-BQ (0.252%, wt%) and G-BN (0.048%, wt%) in THF.

Ion conductive solution: PMMA (60.2%, wt%), TBAPF_6_ (25.1%, wt%) and 1,4-dutyrolactone (14.7%, wt%) in THF.

Ion storage solution: PTMA-co-BP (10 mg/mL) in THF.

As shown in **Scheme S5**, first, the EFC layer was deposited by drop coating on the first ITO glass. Next, the ion storage film was deposited by spin coating on the second ITO glass. Then, the ion storage layer was obtained from UV-crosslinking in the glove box (254 nm for 10 min). Then, the ion conductive film was obtained by continuing to drop the ion conductive solution on the ion-storage film. Finally, three-layer EFC device was fabricated by assembling the two ITO glasses together. The whole process took place in the glove box.


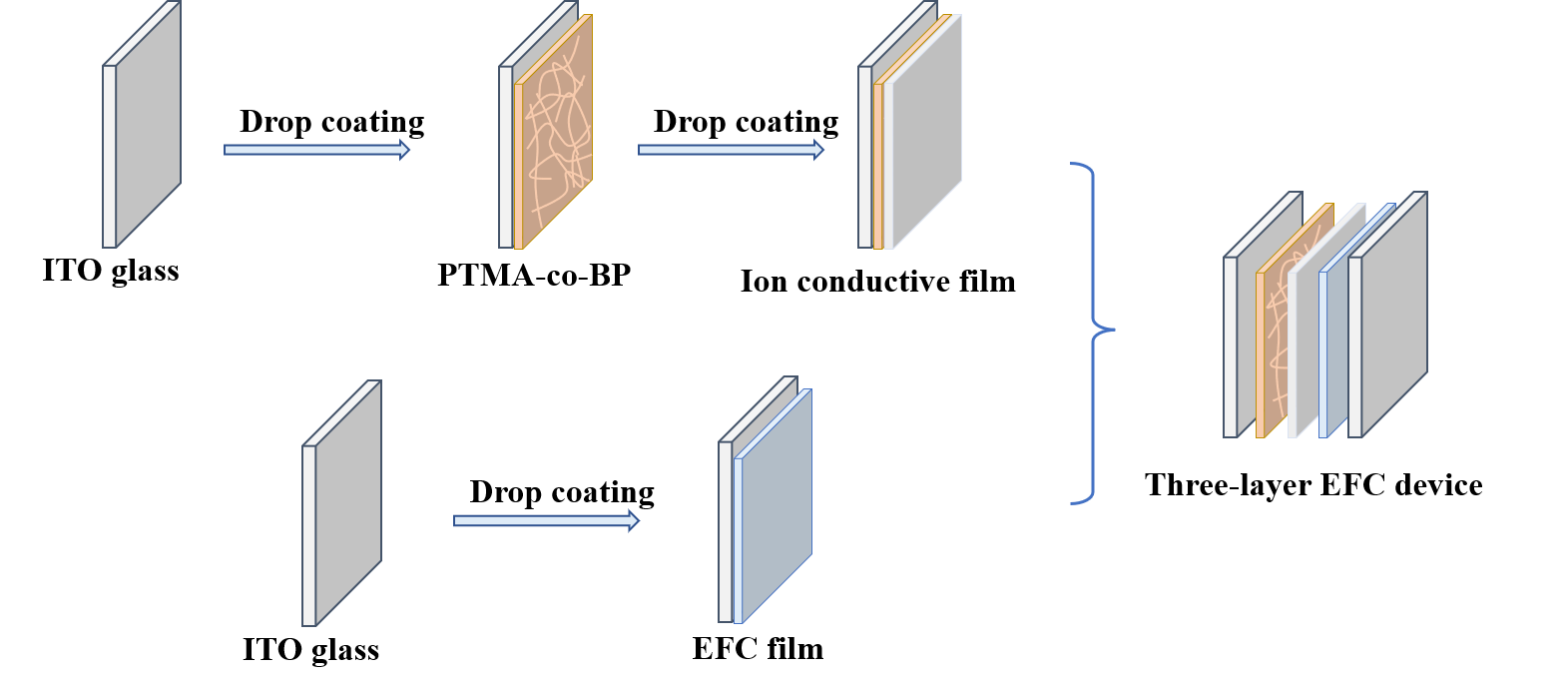


**Scheme S5.** Fabrication process of three-layer EFC device.

**Fabrication of devices with B-BN as EFC material**

**Two-layer EFC devices**

EFC solution: PMMA (24.7%, wt%), TBAPF_6_ (1.1%, wt%), 1,4-dutyrolactone (73.9%, wt%), electro-Lewis base (*p*-BQ) (0.252%, wt%) and B-BN (0.048%, wt%) in tetrahydrofuran (THF).

Ion storage solution: PTMA-co-BP (10 mg/mL) in THF.

**Fabrication of devices with R-BN as EFC material**

**Two-layer EFC devices**

EFC solution: PMMA (24.7%, wt%), TBAPF_6_ (1.1%, wt%), 1,4-dutyrolactone (73.9%, wt%), electro-Lewis base (*p*-BQ) (0.206%, wt%) and R-BN (0.094%, wt%) in anisole.

Ion storage solution: PTMA-co-BP (10 mg/mL) in THF.

# Notes


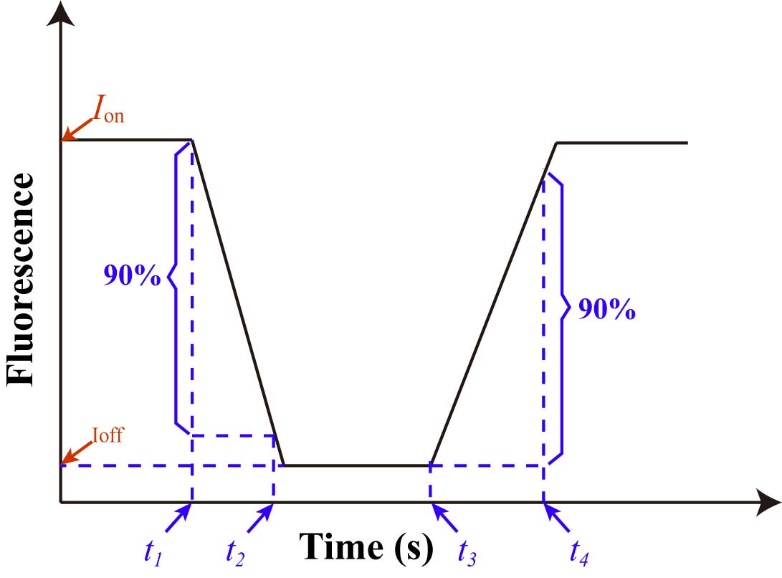


**Scheme S6.** Diagram of fluorescence change under the voltage stimulation.

The fluorescence quenching efficiency (*ŋ*, %), calculated at λ_max_ by Equation (1):

$\eta=\frac{I_{\mathrm{on}}-I_{\mathrm{off}}}{I_{\mathrm{on}}}\times100\%$  (1)

The switching time, which is the time at a 90% of the full fluorescence change occurs after applying potential. It can be calculated by Equation (2) or Equation (3):

$t_{\mathrm{on}}=t_{2}-t_{1}$ (2)

$t_{\mathrm{off}}=t_{4}-t_{3}$ (3)

# Figures

**
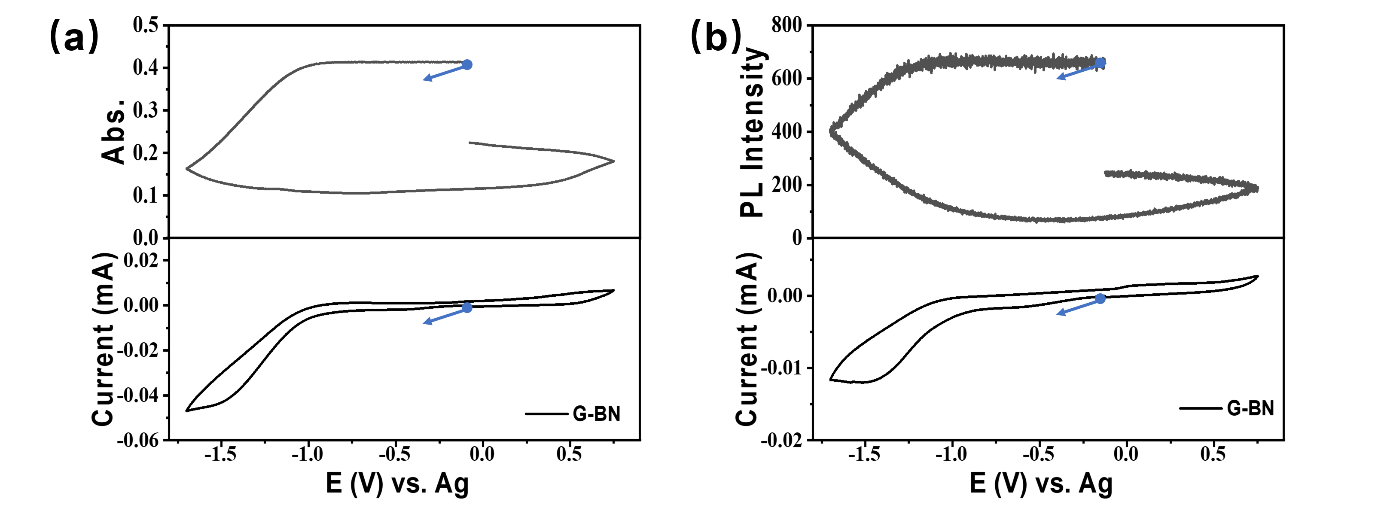
**

**Figure S1.** Changes in (a) absorption spectra at 474 nm (top) and (b) emission spectra at 498 nm (top) during in situ cyclic voltammograms (CVs, bottom) of G-BN (1.0 × 10^–4^ mol L^–1^) in THF with 1.0 × 10^–1^ mol L^–1^ tetrabutylammonium hexafluorophosphate (TBAPF_6_), ex = 465 nm.


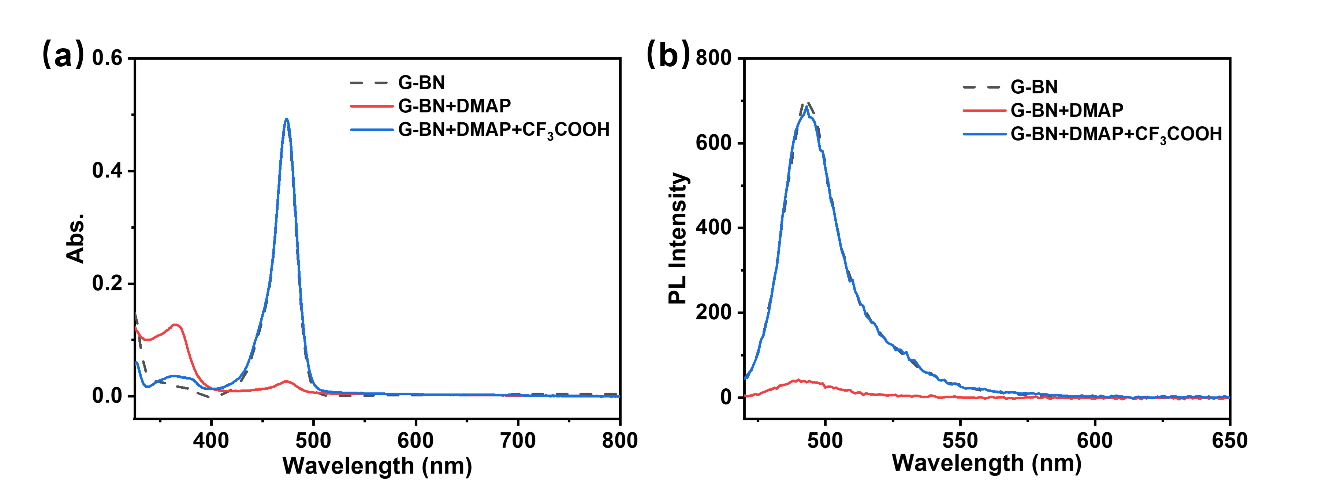


**Figure S2.** Reversible (a) absorption and (b) emission spectra of G-BN (1.0× 10^–5^ mol L^–1^) in THF with the addition of DMAP (2.0 × 10^–3^ mol L^–1^) and CF_3_COOH (3.0 × 10^–3^ mol L^–1^), ex = 465 nm.


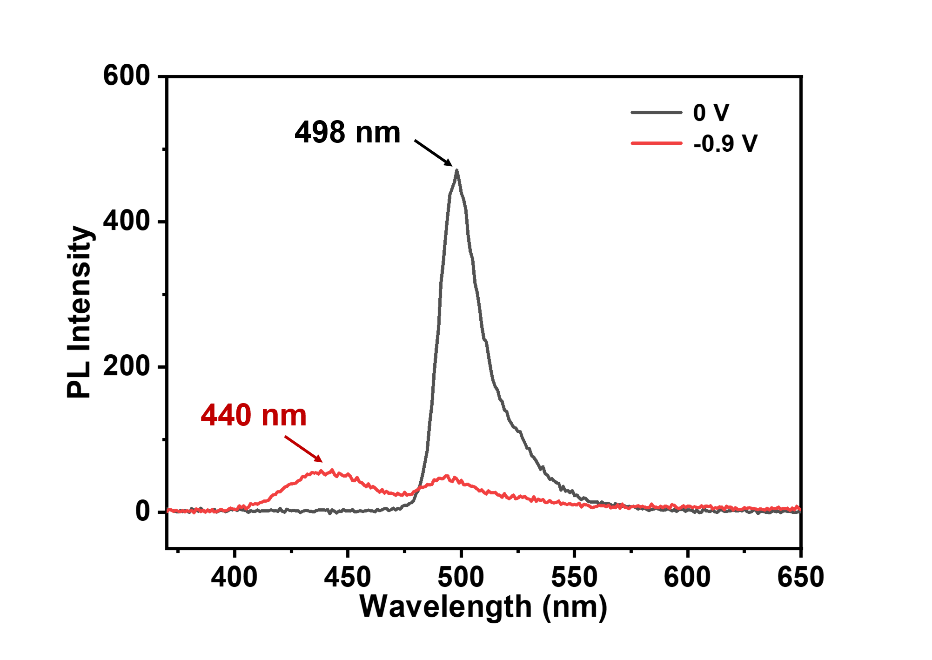


**Figure S3.** The emission spectra of the mixture of *p*-BQ (1.0 × 10^–3^ mol L^–1^) and G-BN (1.0 × 10^–4^ mol L^–1^) in THF with 1.0 × 10^–1^ mol L^–1^ TBAPF_6_ when the solutions were added 0 V and –0.9 V in situ, respectively.


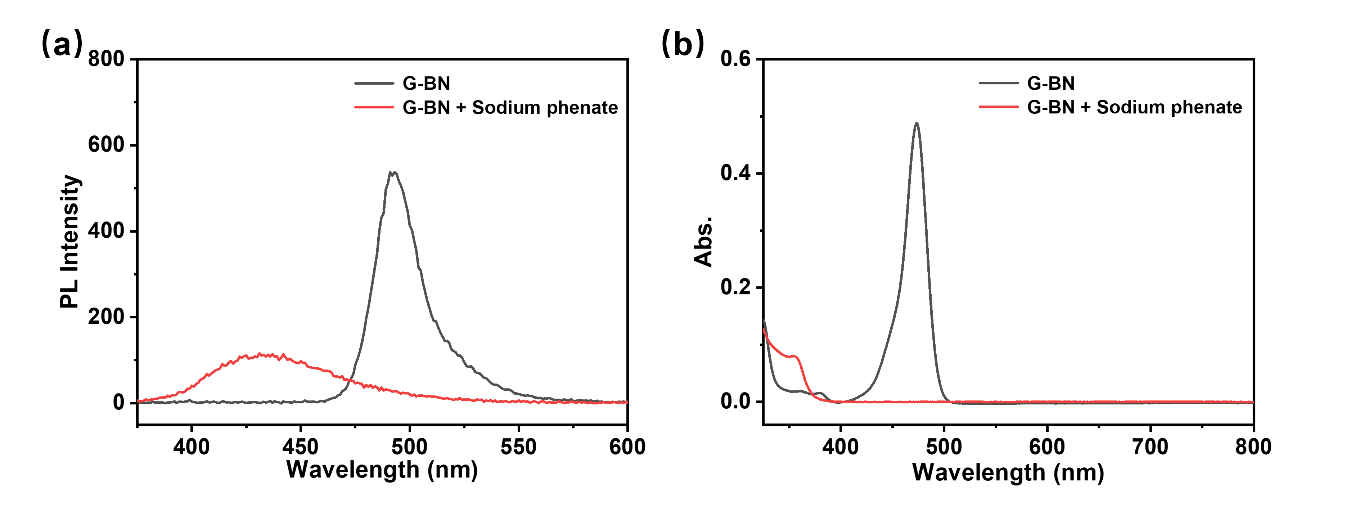


**Figure S4.** Reversible (a) emission and (b) absorbance spectra of G-BN (1.0× 10^–5^ mol L^–1^) in THF with the addition of sodium phenate (2.0 × 10^–5^ mol L^–1^), ex = 355 nm.


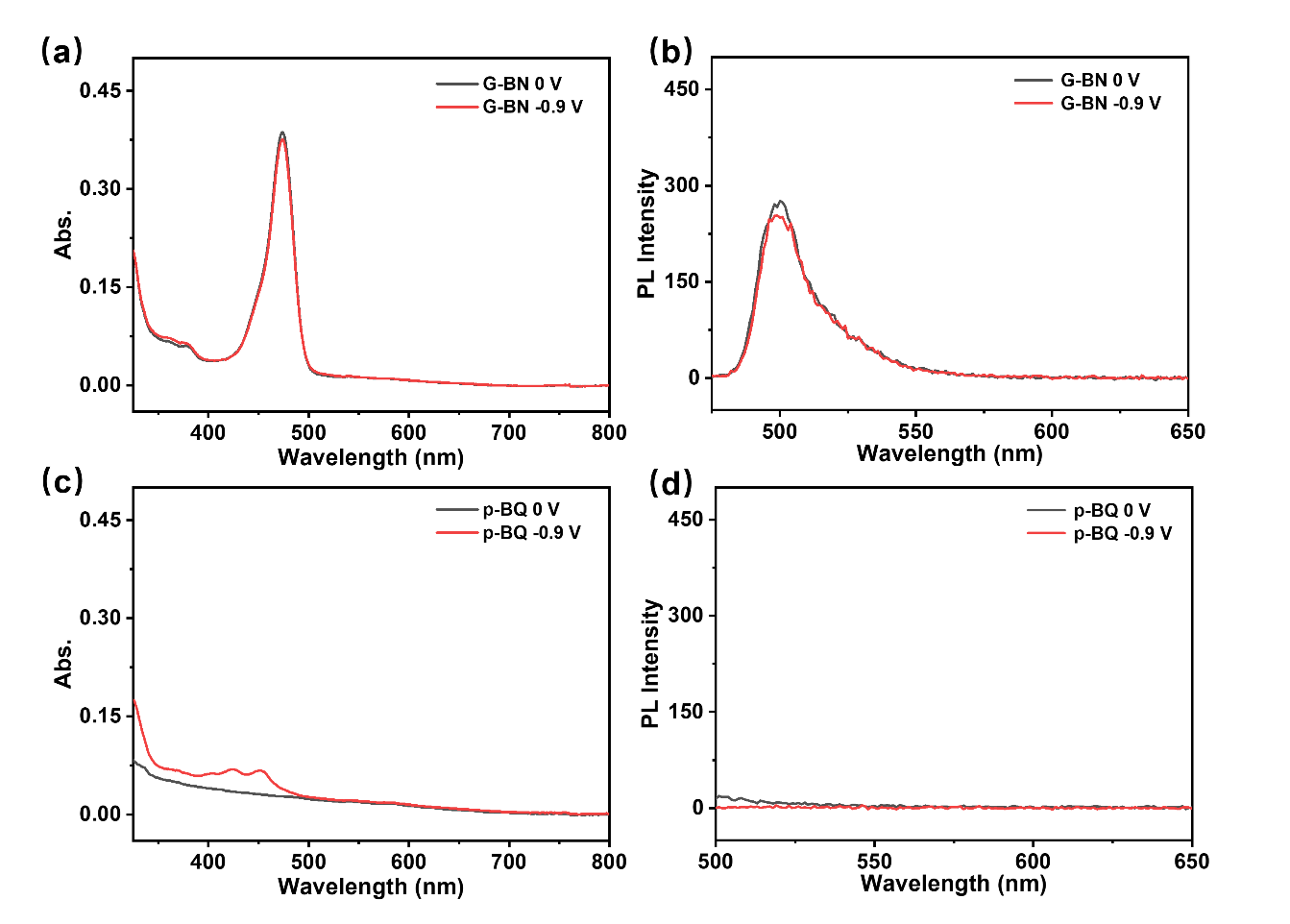


**Figure S5.** The spectra of (a and c) absorption and (b and d) emission spectra of alone G-BN (1.0 × 10^–4^ mol L^–1^) or *p*-BQ (1.0 × 10^–3^ mol L^–1^) in THF with 1.0 × 10^–1^ mol L^–1^ TBAPF_6_ when the solutions were added 0 V and –0.9 V in situ, ex = 465 nm.


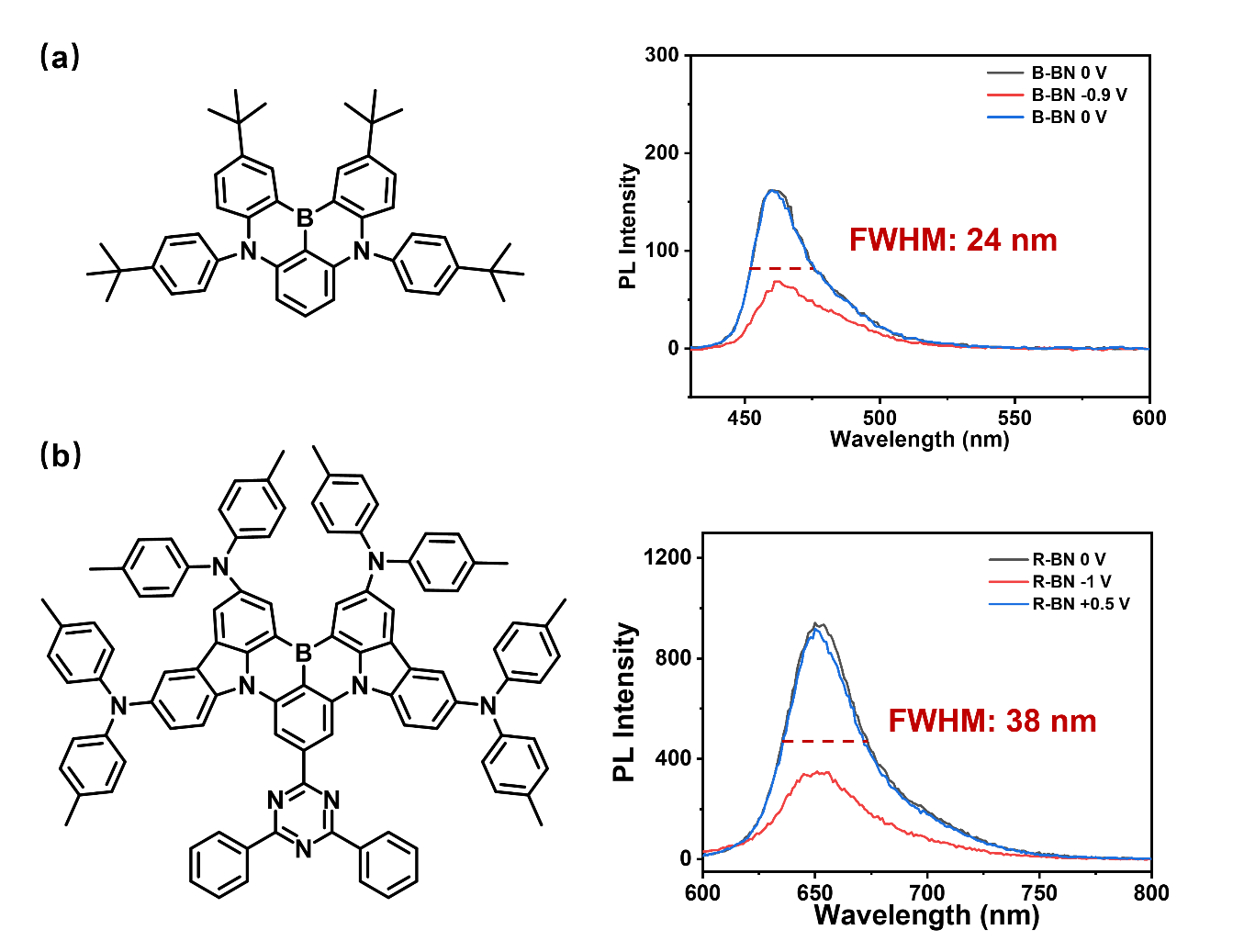


**Figure S6.** Molecular structure of (a) B-BN and (b) R-BN, and emission spectra of the mixture of (a) B-BN (1.0 × 10^–4^ mol L^–1^) and (b) R-BN (1.0 × 10^–4^ mol L^–1^) mixed with *p*-BQ (1.0 × 10^–3^ mol L^–1^) and in THF with 1.0 × 10^–1^ mol L^–1^ TBAPF_6_ when the solutions were added electric stimulation in situ, respectively.


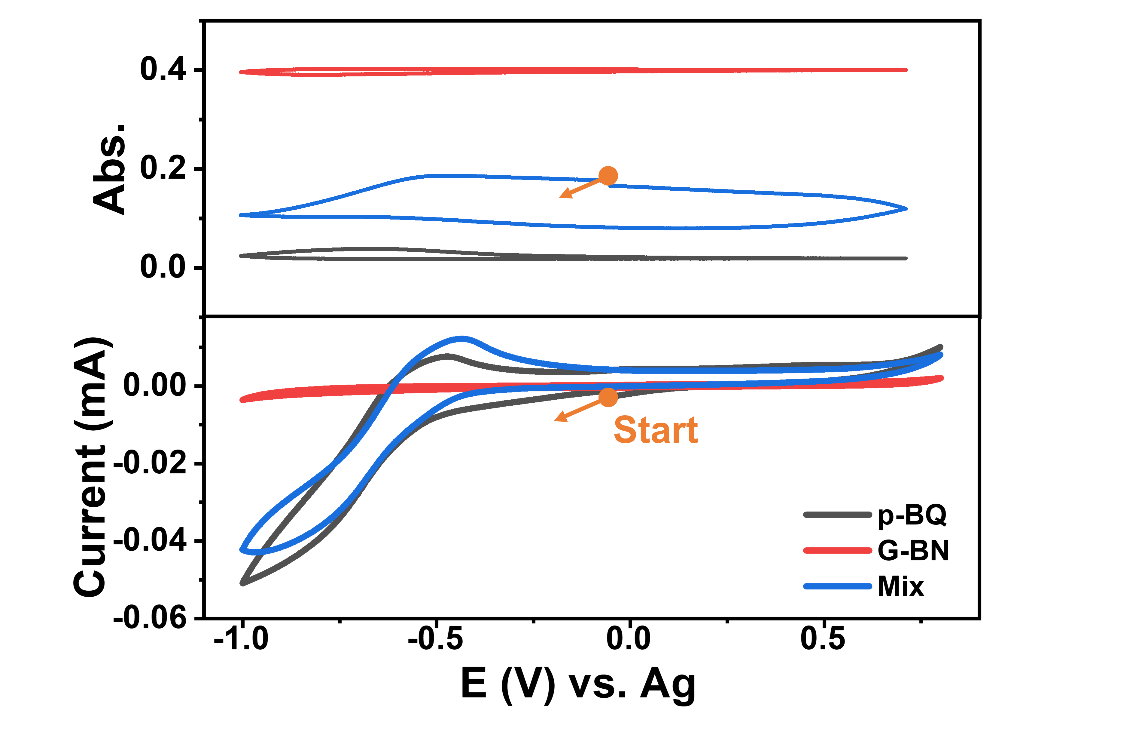


**Figure S7.** Changes in absorption spectra at 474 nm (top) during cyclic voltammograms (CVs, bottom) in situ of G-BN (1.0 × 10^–4^ mol L^–1^), *p*-BQ (1 × 10^–3^ mol L^–1^), and the mixture (mix) of G-BN and *p*-BQ (1.0 × 10^–4^ mol L^–1^ and 1.0 × 10^–3^ mol L^–1^) in THF with 1.0 × 10^–1^ mol L^–1^ TBAPF_6_.


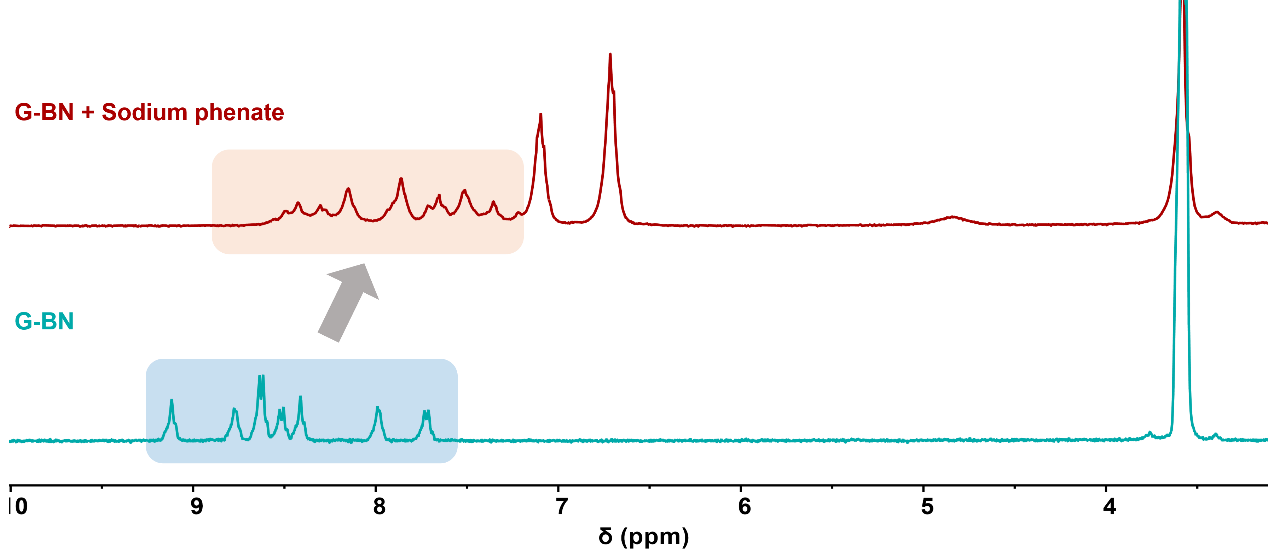


**Figure S8.** ^1^H-NMR spectra of G-BN and the mixture of DtBuCzB and excess sodium phenolate in THF-*d_8_* recorded at 400 MHz at room temperature.


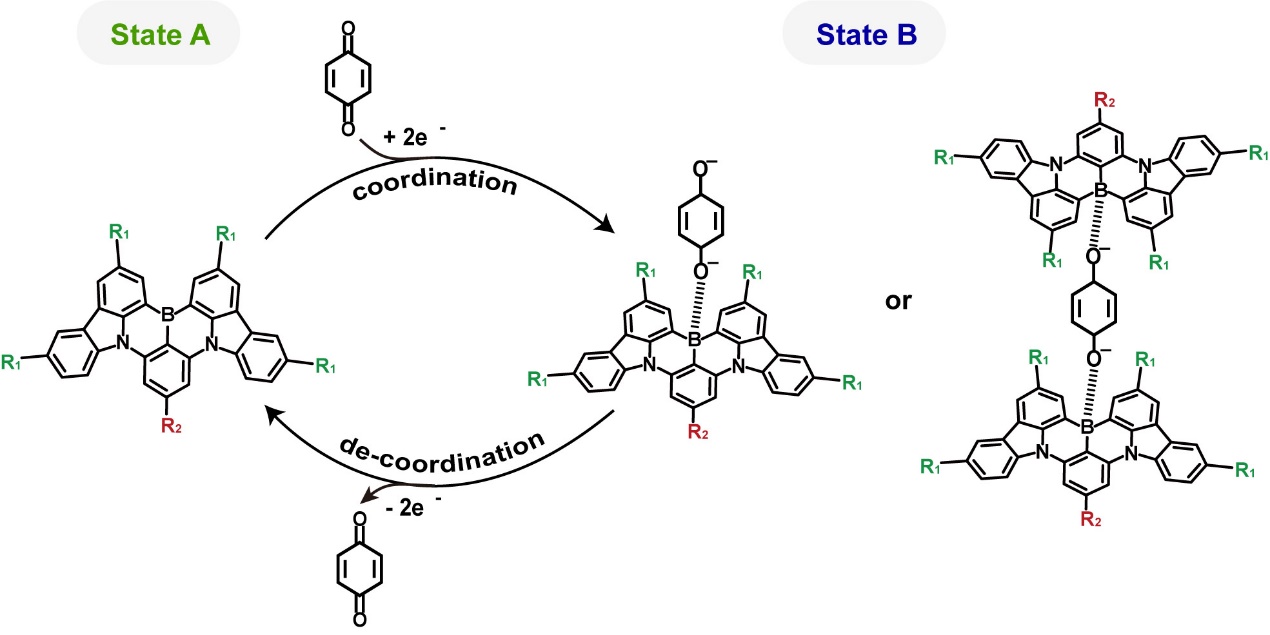


**Figure S9.** Schematic diagram of electrofluorochromic mechanism.


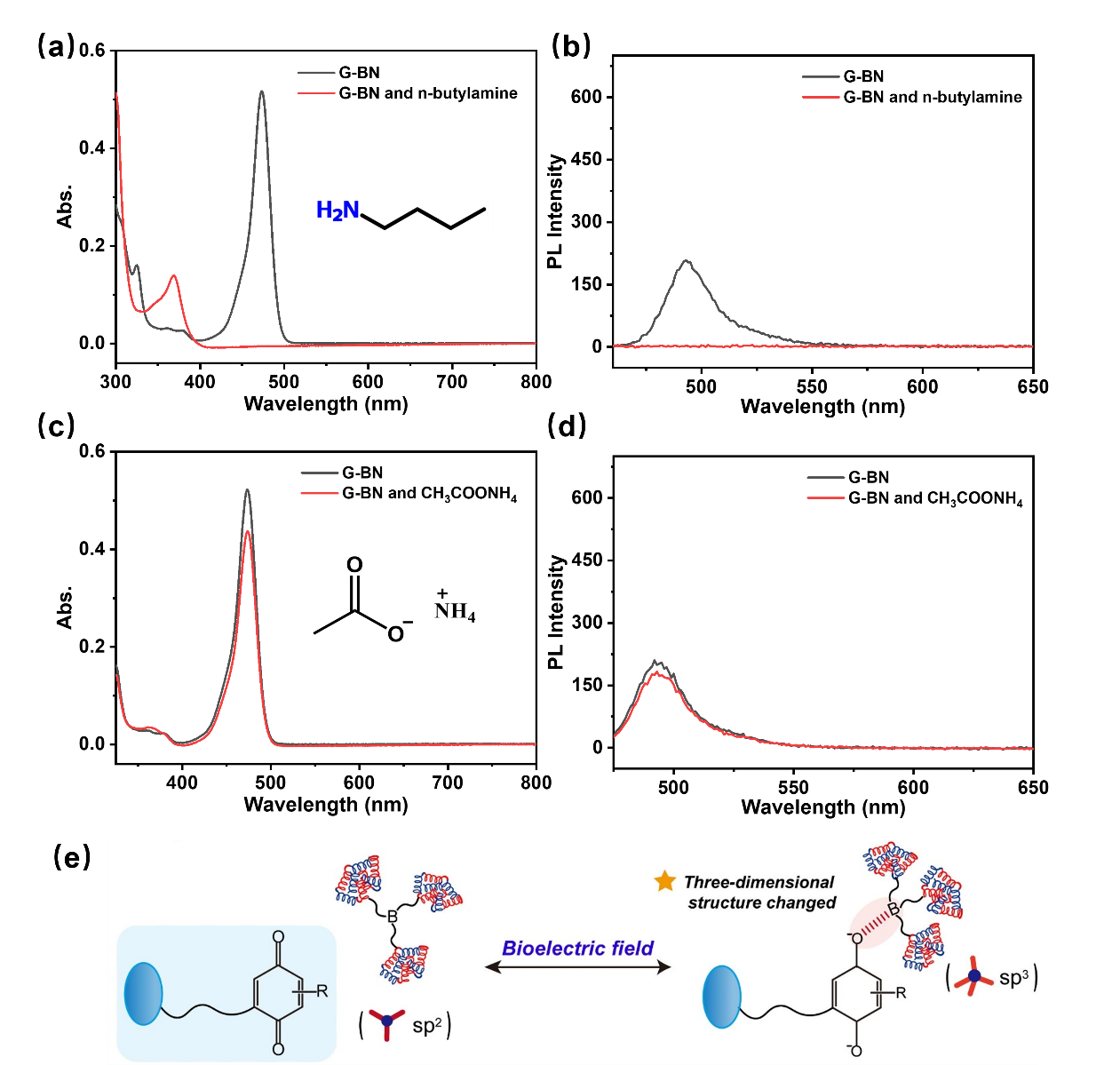


**Figure S10.** The spectra of (a) absorption and (b) emission of alone G-BN (1.0 × 10^–5^ mol L^–1^) and the mixture of G-BN (1.0 × 10^–5^ mol L^–1^) and n-butylamine (1.0 × 10^–4^ mol L^–1^) in THF. The spectra of (c) absorption and (b) emission of alone G-BN (1.0 × 10^-5^ mol L^-1^) and the mixture of G-BN (1.0 × 10^–5^ mol L^–1^) and CH_3_COONH_4_ (2.0 × 10^–4^ mol L^–1^) in THF. (e) Schematic diagram of reflection for electrochemically controlled structure/function-alteration on biological process.


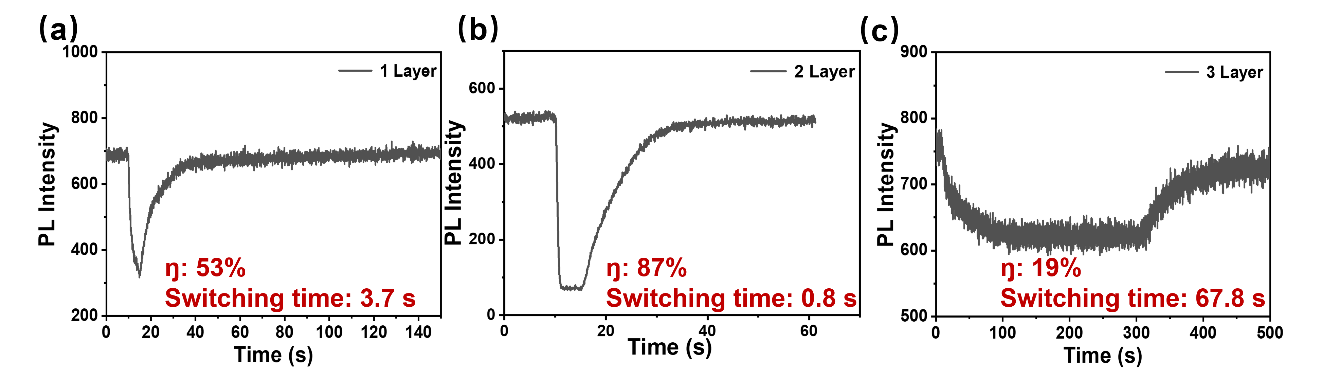


**Figure S11.** Switching properties of EFC devices with different structure: (a) one-layer gel device, (b) two-layer gel device, and (c) three-layer semi-solid device at 498 nm, ex = 465 nm.


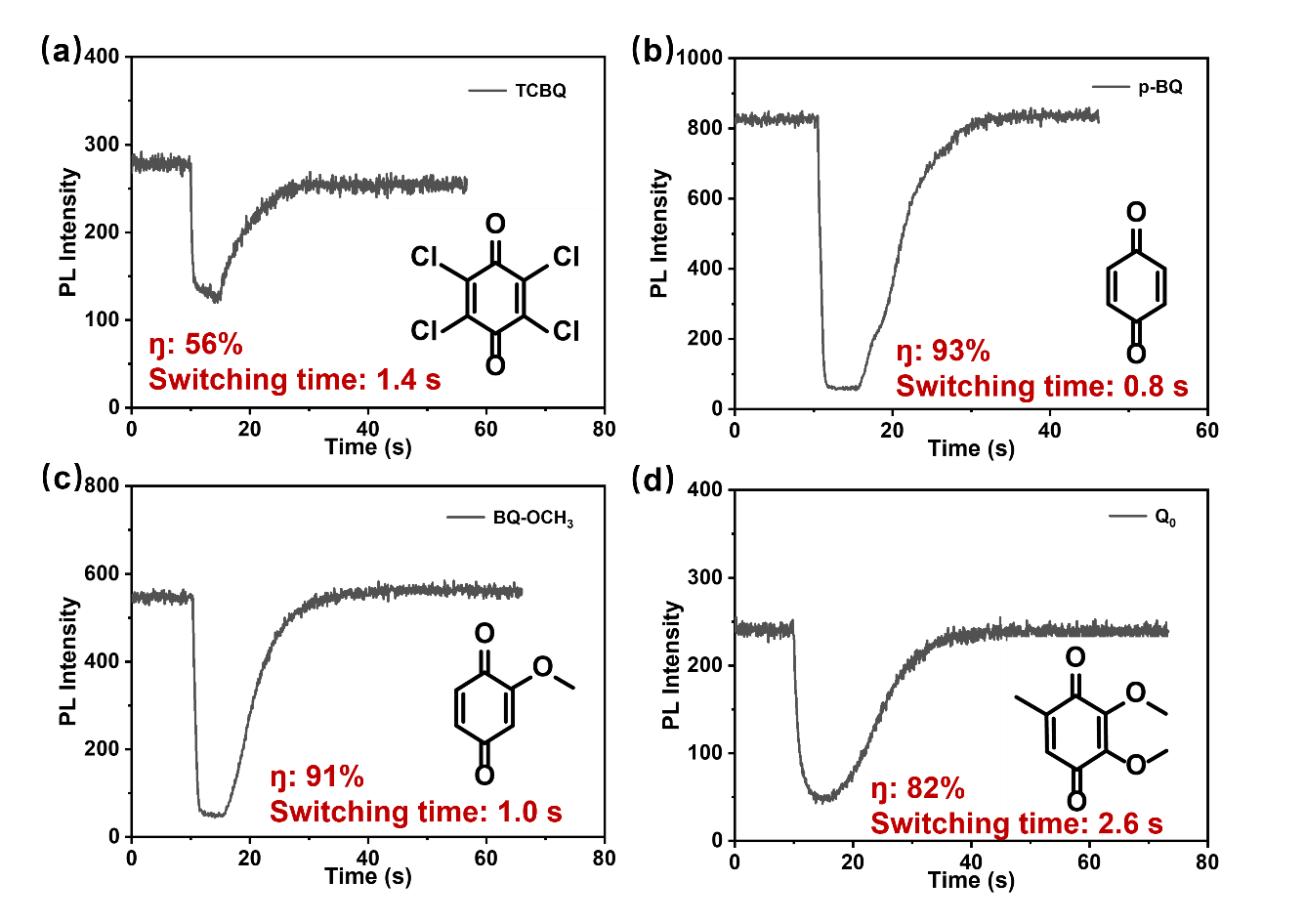


**Figure S12.** Switching properties of EFC devices with (a) TCBQ, (b) *p*-BQ, (c) BQ-OCH_3_ and (d) Q_0_ as ‘electro-Lewis base’ at 498 nm, ex = 465 nm.


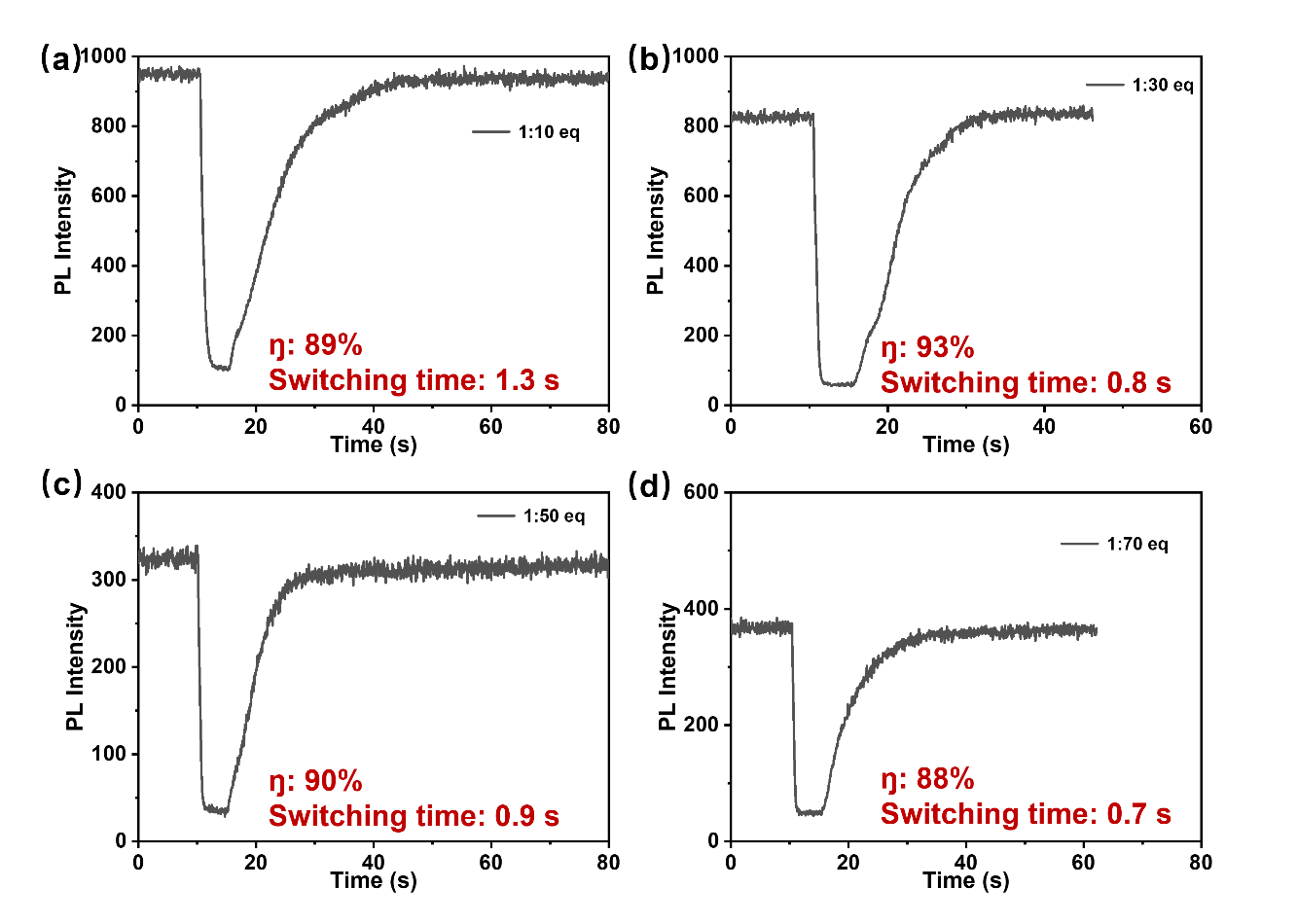


**Figure S13.** Switching properties of EFC devices with different equivalents of G-BN relative to *p*-BQ at 498 nm, ex = 465 nm: (a) 1:10 eq., (b) 1:30 eq., (c) 1:50 eq., and (d) 1:70 eq.


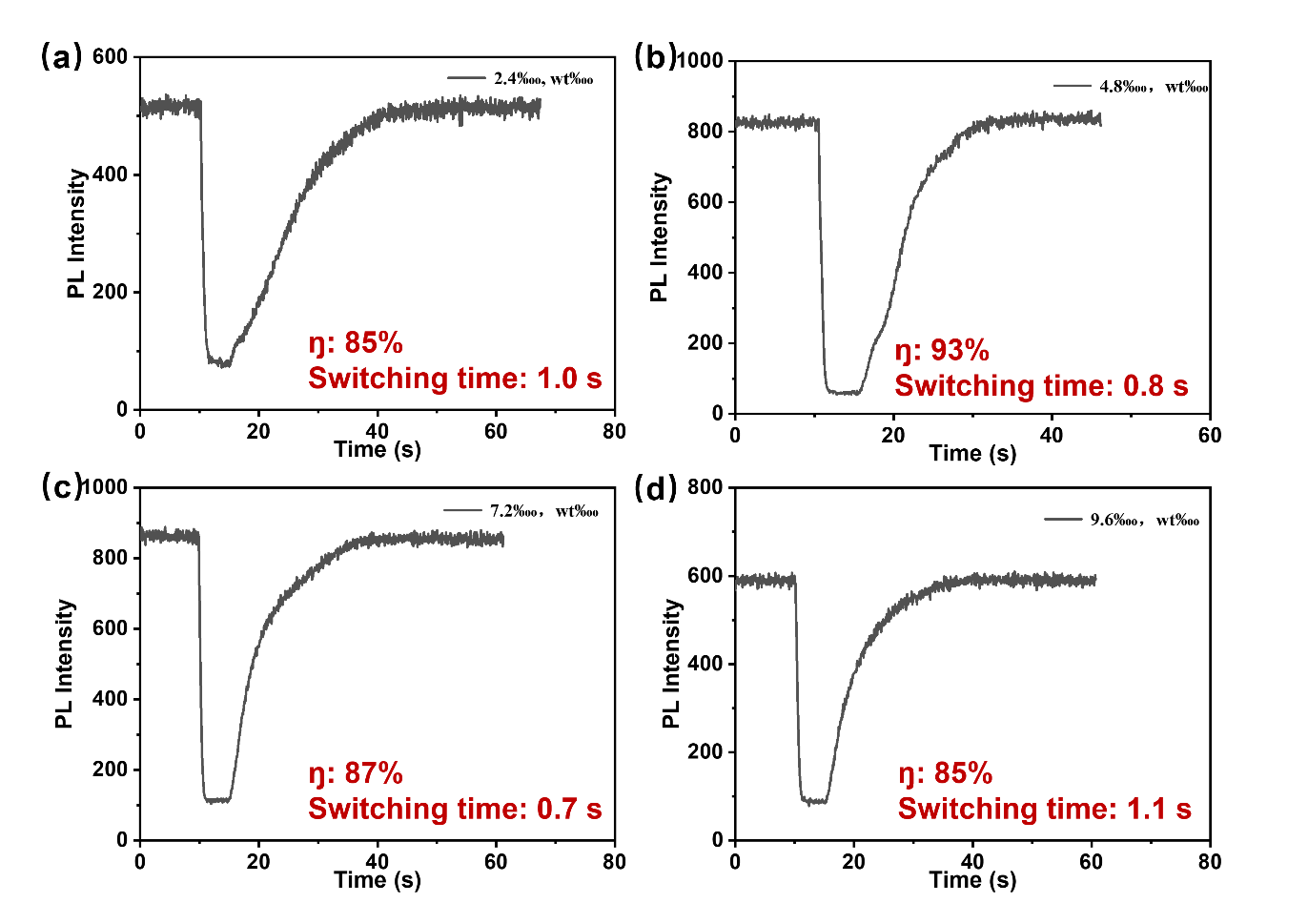


**Figure S14.** Switching properties of EFC devices with different mass percentage of G-BN (wt‱) at 498 nm, ex = 465 nm: (a) 2.4‱, (b) 4.8‱, (c) 7.2‱, and (d) 9.6‱.


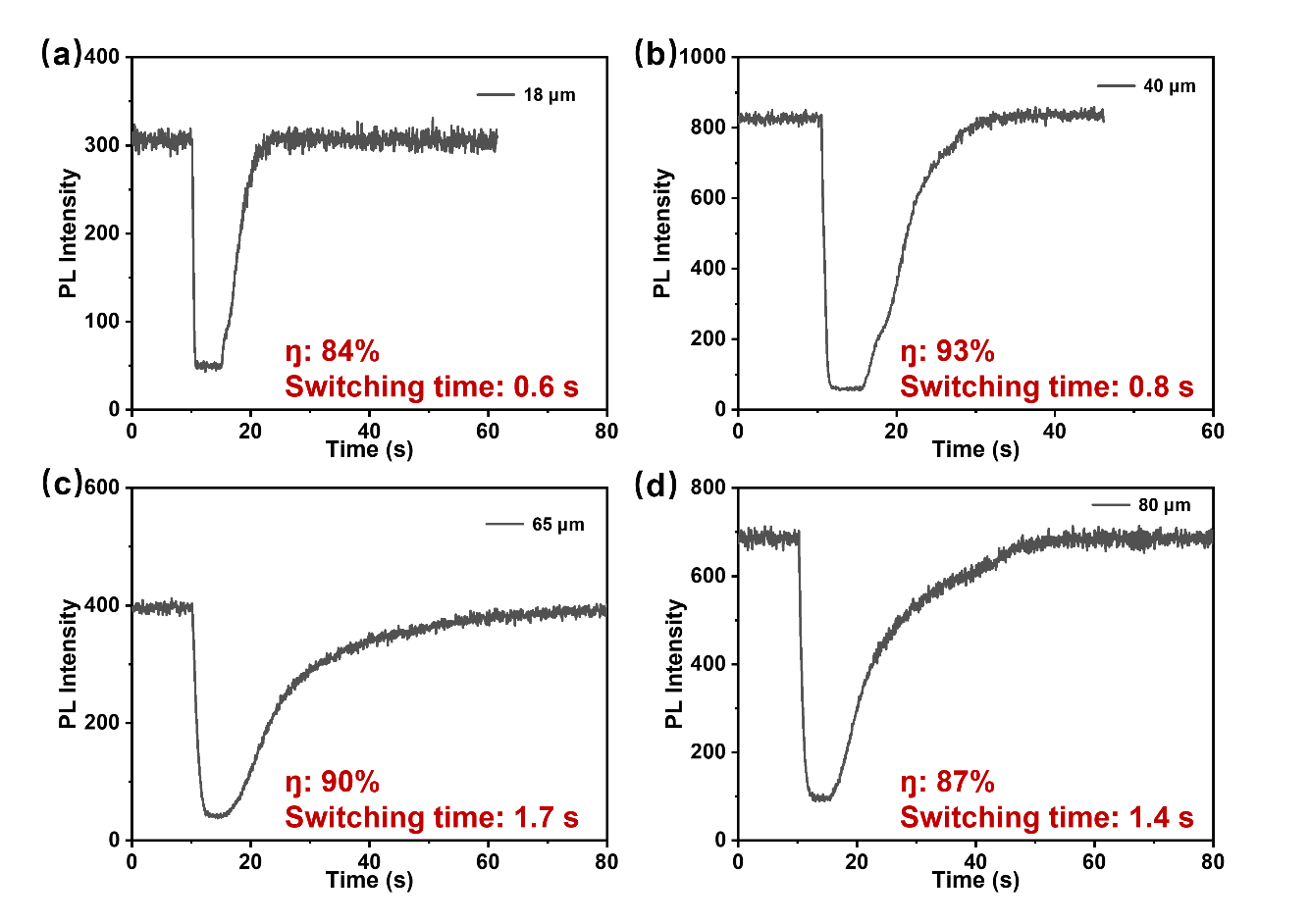


**Figure S15.** Switching properties of EFC devices with different thicknesses of EFC layer at 498 nm, ex = 465 nm: (a) 18 μm, (b) 40 μm, (c) 65 μm and (d) 80 μm.


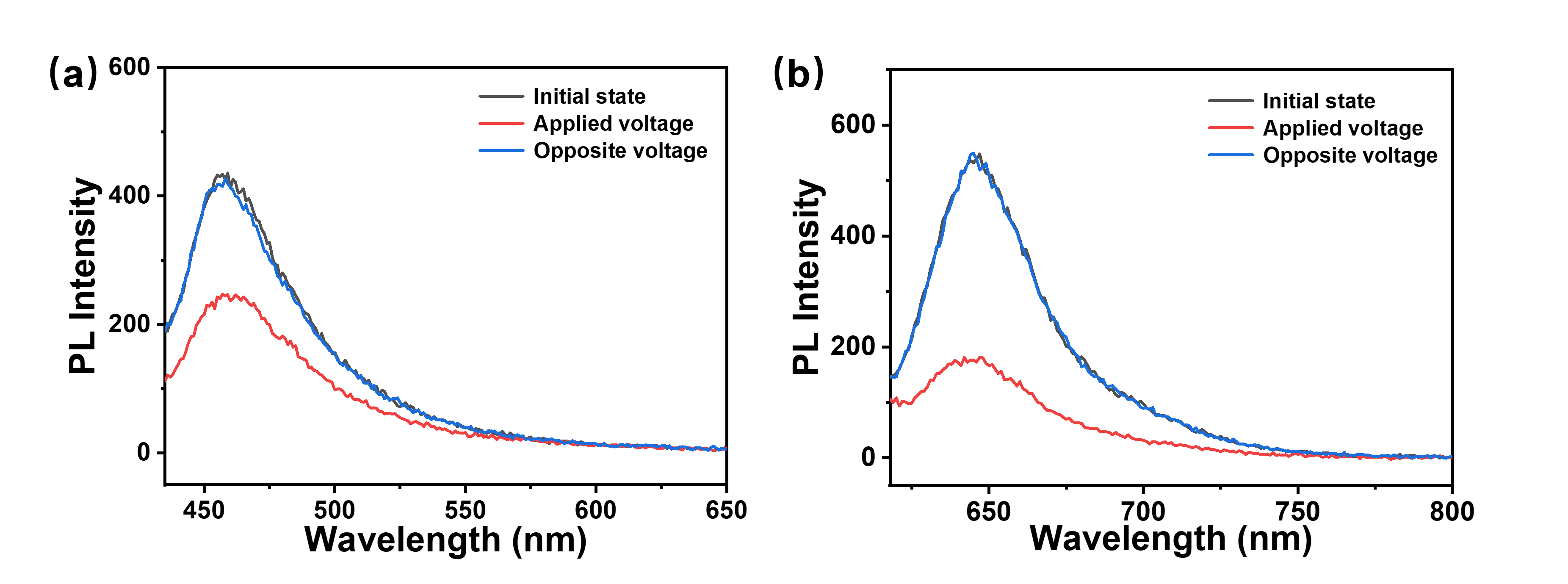


**Figure S16.** Reversible fluorescence switch of the device with (a) B-BN and (b) R-BN as EFC material in emission spectra when added voltages.

**
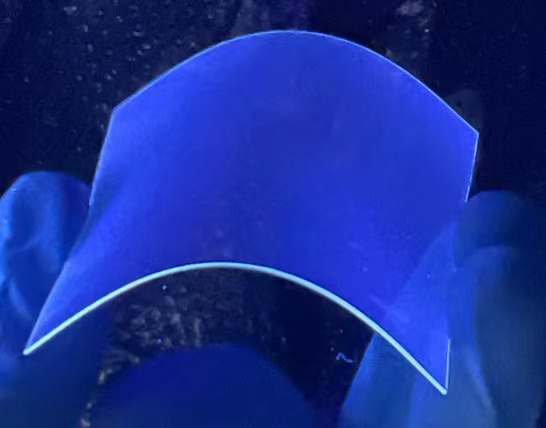
**

**Figure S17.** The intrinsic fluorescence of PET-ITO under a UV light at 365 nm.


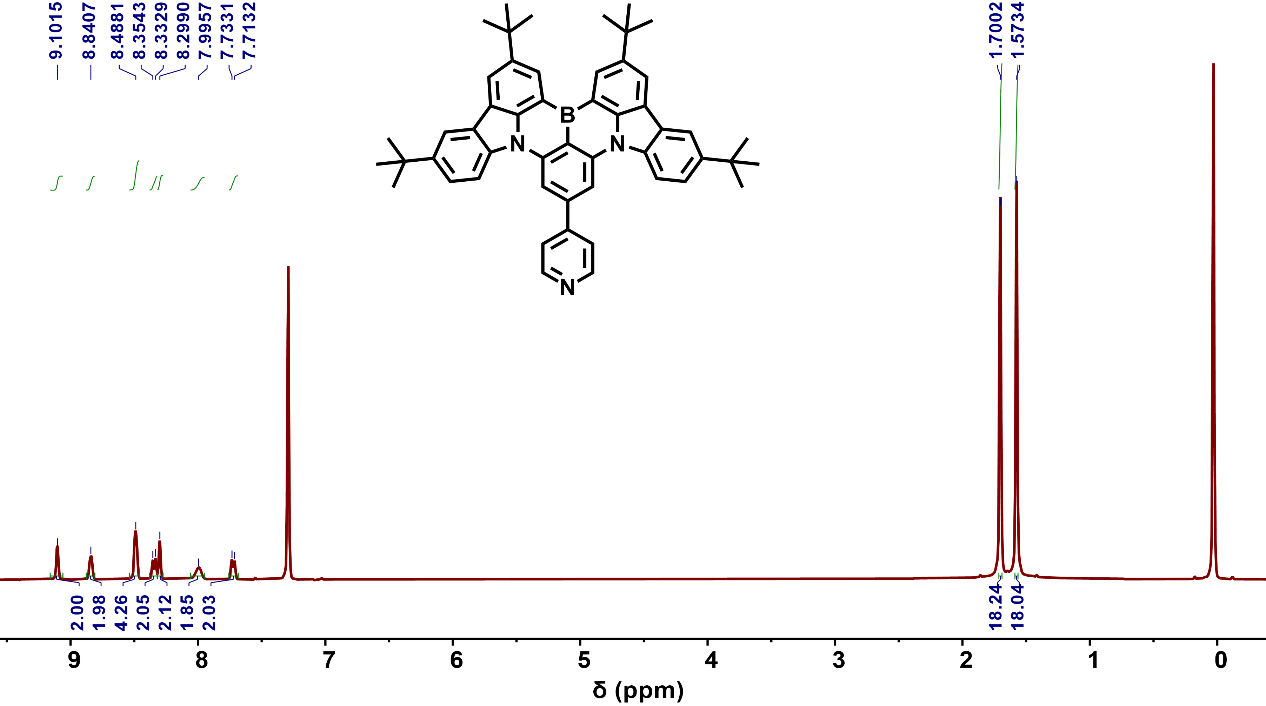


**Figure S18.** ^1^H-NMR spectra of G-BN in CDCl_3_ recorded at 400 MHz at room temperature.

# Tables

**Table S1.** Brief summary of several well-known electrofluorochromic devices.

| **EFC Materials** | **FWHM** | **t_on_** | **t_off_** | **Refs** |
| --- | --- | --- | --- | --- |
|  | **30 nm** | **0.6 s** | **2.4 s** | **This work** |
|  | 80 nm | 2.7 s | 7.5 s | [S2] |
|  | 90 nm | 9.5 s | 16.4 s | [S3] |
|  | 100 nm | 0.25 s | 4.42 s | [S4] |
|  | 110 nm | 0.4 s | 38 s | [S5] |
|  | - | 2 s | 7 s | [S6] |
|  | - | 5 s | 10 s | [S7] |
|  | 45 nm | - | - | [S8] |
|  | 50 nm | 2 s | 2 s | [S9] |
|  | 55 nm | >30 s | | [S10] |
|  | >40 nm | - | - | [S11] |
|  | 35 nm | - | - | [S12] |
|  | 70 nm | - | - | [S13] |
|  | 60 nm | 5 s | 20 s | [S14] |
|  | 50 nm | 8.1 s | 48.3 s | [S15] |
|  | 100 nm | 0.7 s | 116.9 s |  |
|  | 160 nm | 3.6 s | 3.5 s | [S16] |
|  | 100 nm | - | - | [S17] |
|  | 70 nm | - | - | [S18] |
|  | 40 nm | - | - | [S19] |
|  | 80 nm | - | - | [S20] |
|  | 170 nm | 47 s | 20 s | [S21] |
|  | 60 nm | 0.97 s | 0.76 s | [S22] |
|  | 40 nm | 0.72 s | 0.76 s |  |
|  | 70 nm | 2.05 s | 3.36 s |  |

**Table S2.** The summary of physical properties of **G-BN** (sp^2^-hybrid), **G-BN·OC_6_H_5_^–^** (sp^3^-hybrid) in solution.

| **Solution** | Ф_PL, ex=450 nm_ | Ф_PL, ex=355 nm_ | τ_p_ [ns] |
| --- | --- | --- | --- |
| **G-BN** | 94.71% (493 nm) | - | 5.21 |
| **G-BN·OC_6_H_5_^–^** | 0.38% (493 nm) | 10.77% (433 nm) | 3.75 |

# The References

1. B. Yang, Y.-M. Zhang, C. Wang, C. Gu, Y. Yan, G. Yang, H. Yin, C. Li, S. X.-A. Zhang, *Nat. Commun.* **2024**, *15*, 5166.
2. H.-T. Lin, J.-T. Wu, M.-H. Chen, G.-S. Liou, *J. Mater. Chem. C* **2020,** *8*, 12656.
3. H. T. Lin, C. L. Huang, G. S. Liou, *ACS Appl. Mater. Interfaces* **2019,** *11*, 11684.
4. J. Sun, Z. Liang, *ACS Appl. Mater. Interfaces* **2016,** *8*, 18301.
5. J.-H. Wu, G.-S. Liou, *Adv. Funct. Mater.* **2014,** *24*, 6422.
6. Y. Kim, H. Ohmagari, A. Saso, N. Tamaoki, M. Hasegawa, *ACS Appl. Mater. Interfaces* **2020,** *12*, 46390.
7. K. Kanazawa, Y. Komiya, K. Nakamura, N. Kobayashi, *Phys. Chem. Chem. Phys.* **2017***, 19,* 16979.
8. M. Dias, P. Hudhomme, E. Levillain, L. Perrin, Y. Sahin, F.-X. Sauvage, C. Wartelle, *Electrochem. Commun.* **2004,** *6*, 325.
9. Y. Kim, J. Do, E. Kim, G. Clavier, L. Galmiche, P. Audebert, *J. Electroanal. Chem.* **2009,** *632*, 201.
10. S. Seo, Y. Kim, Q. Zhou, G. Clavier, P. Audebert, E. Kim, *Adv. Funct. Mater.* **2012,** *22*, 3556.
11. H. Lim, S. Seo, S. Pascal, Q. Bellier, S. Rigaut, C. Park, H. Shin, O. Maury, C. Andraud, E. Kim, *Sci. Rep.* **2016,** *6*, 18867.
12. K. Tsujimoto, R. Ogasawara, T. Nakagawa, H. Fujiwara, *Eur. J. Inorg. Chem.* **2014,** *2014*, 3960.
13. N. L. Bill, J. M. Lim, C. M. Davis, S. Bahring, J. O. Jeppesen, D. Kim, J. L. Sessler, *Chem. Commun.* **2014,** *50*, 6758.
14. K. Kanazawa, K. Nakamura, N. Kobayashi, *Sol. Energy Mater. Sol. Cells* **2016,** *145*, 42.
15. M. Chang, W. Chen, H. Xue, D. Liang, X. Lu, G. Zhou, *J. Mater. Chem. C* **2020,** *8*, 16129.
16. A. Beneduci, S. Cospito, M. La Deda, L. Veltri, G. Chidichimo, *Nat. Commun.* **2014,** *5*, 3105.
17. R. A. Illos, D. Shamir, L. J. W. Shimon, I. Zilbermann, S. Bittner, *Tetrahedron Lett.* **2006,** *47*, 5543.
18. Y.-X. Yuan, Y. Chen, Y.-C. Wang, C.-Y. Su, S.-M. Liang, H. Chao, L.-N. Ji, *Inorg. Chem. Commun.* **2008,** *11*, 1048.
19. M. Čížková, L. Cattiaux, J. Pandard, M. Guille-Collignon, F. Lemaître, J. Delacotte, J.-M. Mallet, E. Labbé, O. Buriez, *Electrochem. Commun.* **2018,** *97*, 46.
20. O. Galangau, I. Fabre-Francke, S. Munteanu, C. Dumas-Verdes, G. Clavier, R. Méallet-Renault, R. B. Pansu, F. Hartl, F. Miomandre, *Electrochim. Acta* **2013,** *87*, 809.
21. G. A. Corrente, F. Parisi, V. Maltese, S. Cospito, D. Imbardelli, M. La Deda, A. Beneduci, *Molecules* **2021,** *26*, 6818.
22. X. Wang, W. Li, W. Li, C. Gu, H. Zheng, Y. Wang, Y. M. Zhang, M. Li, S. X.-A. Zhang, *Chem. Commun.* **2017,** *53*, 11209.
